# Mechanism of ribosome rescue by alternative ribosome-rescue factor B

**DOI:** 10.1038/s41467-020-17853-7

**Published:** 2020-08-14

**Authors:** Kai-Hsin Chan, Valentyn Petrychenko, Claudia Mueller, Cristina Maracci, Wolf Holtkamp, Daniel N. Wilson, Niels Fischer, Marina V. Rodnina

**Affiliations:** 1 Department of Physical Biochemistry, Max Planck Institute for Biophysical Chemistry, Am Fassberg 11, 37077 Göttingen, Germany; 2 Department of Structural Dynamics, Max Planck Institute for Biophysical Chemistry, Am Fassberg 11, 37077 Göttingen, Germany; 3 Institute for Biochemistry and Molecular Biology, University of Hamburg, Martin-Luther-King-Platz 6, 20146 Hamburg, Germany; 4 Present Address: Paul-Ehrlich-Institut, Paul-Ehrlich-Straße 51-59, 63225 Langen, Germany

**Keywords:** Kinetics, Ribosome, Cryoelectron microscopy

## Abstract

Alternative ribosome-rescue factor B (ArfB) rescues ribosomes stalled on non-stop mRNAs by releasing the nascent polypeptide from the peptidyl-tRNA. By rapid kinetics we show that ArfB selects ribosomes stalled on short truncated mRNAs, rather than on longer mRNAs mimicking pausing on rare codon clusters. In combination with cryo-electron microscopy we dissect the multistep rescue pathway of ArfB, which first binds to ribosomes very rapidly regardless of the mRNA length. The selectivity for shorter mRNAs arises from the subsequent slow engagement step, as it requires longer mRNA to shift to enable ArfB binding. Engagement results in specific interactions of the ArfB C-terminal domain with the mRNA entry channel, which activates peptidyl-tRNA hydrolysis by the N-terminal domain. These data reveal how protein dynamics translate into specificity of substrate recognition and provide insights into the action of a putative rescue factor in mitochondria.

## Abstract (web summary)

Rescue of ribosomes stalled on non-stop mRNA is essential for cell viability, and several rescue systems to resolve stalling exist in bacteria. Here, the authors use rapid kinetics and cryo-EM to reveal the pathway and selectivity mechanism of ArfB-mediated ribosome rescue.

## Introduction

Rescue of ribosomes stalled on nonstop mRNAs is essential for most bacterial species^1^, and for mitochondrial translation in humans^2,3^. The lack of a stop codon causes ribosomes to stall at the 3′ end of mRNAs, an event that is estimated to occur on 2–4% of translations in *Escherichia coli*^4^. The rescue systems tmRNA-SmpB, ArfA, and ArfB release the truncated translation product, allowing the ribosomes to be recycled for subsequent rounds of translation^5^. Among the rescue systems, only ArfB is able to induce polypeptide release independent of canonical termination factors^6,7^. ArfB is highly conserved among bacteria and has conserved homologs in eukaryotes from yeast to humans^8^. The eukaryotic homolog of ArfB, ICT1, is essential for cell viability and a candidate for nonstop ribosome rescue during translation in mitochondria^2,3^. Given the functional interchangeability and striking structural similarities between ArfB and ICT1^9,10^, studies of ArfB in bacteria may provide insights into how its homolog functions on the mitochondrial ribosome.

The cellular role of ArfB and the molecular mechanism of ArfB-mediated rescue are unclear. Under normal conditions in *E. coli*, ArfB appears redundant with tmRNA-SmpB and ArfA^6^. While tmRNA-SmpB and ArfA preferentially target ribosomes stalled on truncated mRNAs^11–13^, ArfB has been suggested to act on ribosomes stalled on mRNAs extending downstream past the P site^7,14^. If so, one potential function of ArfB could be to rescue ribosomes pausing on rare codon clusters^7^. However, another study indicated that ArfB does not rescue ribosomes stalled on longer mRNAs^3^. We note that the experimental conditions used in the two conflicting reports are similar in that they measured peptidyl-tRNA hydrolysis in vitro after long incubation times^7^, which does not probe the potential kinetic differences in ArfB activity.

The crystal structure of ArfB on the ribosome showed that ArfB consists of N- and C-terminal domains connected by a linker^15^. The globular N-terminal domain contains the conserved GGQ motif essential for catalysis of peptidyl-tRNA hydrolysis^6,7^. Its C-terminal tail, rich in positive residues, forms an α-helix in the mRNA entry channel that would be incompatible with mRNA extending significantly past the P-site codon^15^. In solution, the linker region and at least parts of the C-terminal domain are disordered giving rise to various orientations relative to the N-terminal domain^10^. Intrinsic disorder has been increasingly considered an important factor in understanding the dynamics of molecular recognition^16–18^. However, how the disordered regions of ArfB might contribute to recognition of its cellular targets is not known.

A comprehensive mutagenesis study identified ArfB residues that are important for ribosome binding and peptidyl-tRNA hydrolysis^10^. Remarkably, most of these essential amino acids are not located in the catalytic N-terminal domain, but in the C-terminal tail of ArfB. The crystal structure of ribosome-bound ArfB suggested that the C-terminal domain may function as a sensor for stalled ribosomes by inserting into the empty mRNA entry channel^15^, in apparent agreement with the mutational data. However, a detailed comparison of the structural and mutational data revealed dramatic discrepancies, as nearly all, except one, of the identified essential C-terminal ArfB residues were not involved in ribosome interactions in the crystal structure^10,15^, raising a question as to why these residues are crucial^10^.

In this study, we use a combination of rapid kinetics techniques and cryo-electron microscopy (cryo-EM) to dissect the kinetic and structural mechanism of ArfB action on the ribosome. The results provide insights into the physiologically relevant targets of ArfB, establish a mechanistic model of ArfB-mediated ribosome rescue, and reveal the importance of ArfB dynamics in recognition of stalled ribosome complexes.

## Results

### ArfB rescues ribosomes stalled on short truncated mRNAs

**Fig. 1:**
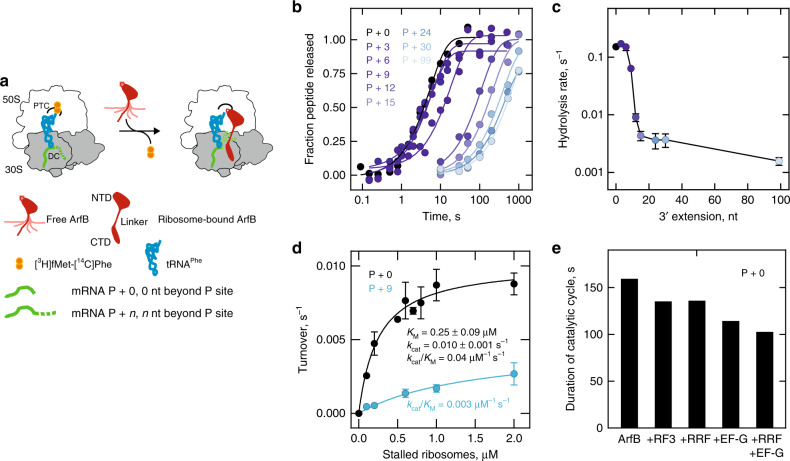
ArfB preferentially rescues ribosomes stalled on short mRNA. **a** Experimental assay for ArfB-mediated ribosome rescue. Ribosome complexes with mRNAs of different length are rapidly mixed with ArfB and the fraction of peptides released from tRNA by ArfB-dependent hydrolysis is quantified. PTC peptidyl transferase center; DC decoding center; NTD N-terminal domain; CTD C-terminal domain. Free ArfB is shown as an ensemble of dynamic molecules^10^, the ribosome-bound ArfB is shown as in the X-ray structure^15^. **b** Time courses of single-round peptidyl-tRNA hydrolysis at excess ArfB (1 µM) over ribosome complexes (0.15 µM). The mRNA length is indicated by number of nucleotides (nt) extending beyond the P site, from none (P + 0) to 99 nt (P + 99). Data represented as mean values of two biological replicates. Solid lines are exponential fits. **c** Rate of hydrolysis at increasing mRNA length. Error bars indicate the SEM of the exponential fits (**b**). **d** Peptidyl-tRNA hydrolysis on P + 0 and P + 9 complexes at limiting ArfB concentrations. Initial velocity of the hydrolysis reaction is measured after mixing ArfB (0.02 µM) with increasing concentrations of P + 0 or P + 9 complexes. Solid lines are results of hyperbolic fitting. Error bars represent the SEM of three biological replicates. **e** Effect of ribosome recycling factors on the duration of an ArfB catalytic cycle on P + 0 complexes (0.2 µM) mixed with catalytic amounts of ArfB (0.02 µM) and excess of RF3 (0.5 µM), RRF (0.5 µM) and EF-G (0.5 µM). All experiments were carried out at 37 °C.

We first used kinetic experiments in a fully reconstituted in vitro system to study whether the activity of ArfB depends on the length of the mRNA in the stalled ribosome complexes. We formed ribosome complexes with mRNAs of different length, let ribosomes translate the first two codons, and then stalled translation by omitting the aminoacyl-tRNA needed to decode the next codon. The resulting complexes contained [^3^H]fMet-[^14^C]Phe-tRNA^Phe^ in the P site, no tRNA in the A site and an mRNA of different lengths (0–99 nucleotides (nt)) extending past the P site, denoted as P + 0 to P + 99 (Fig. 1a). We rapidly mixed these stalled ribosomes with a large excess of purified ArfB and followed the time courses of peptidyl-tRNA hydrolysis by the quench-flow technique (Fig. 1b). When more than 9 nt of the mRNA extended past the P-site codon, the rate of hydrolysis reaction decreases sharply, by about 15-fold (P + 12) to 100-fold (P + 99) (Fig. 1c), indicating that ArfB-mediated rescue is most efficient when the mRNA is short.

In the post-termination complex, the C-terminal domain of ArfB occupies the mRNA entry channel^15^, suggesting that mRNA extending past the P site inhibits ArfB-mediated ribosome rescue. To test whether a long mRNA overhang affects the selectivity of ArfB binding, we measured the steady-state parameters of the rescue pathway at conditions of ArfB turnover, i.e., at catalytic concentrations of ArfB and excess of the ribosomes. We chose the P + 9 construct (i.e., with 9 nt extending past the P site), because this is the mRNA length for which we observe a significant, albeit reduced, ArfB activity. The Michaelis–Menten dependence of initial velocities yields the *k*_cat_/*K*_M_ values of 0.04 µM^−1^ s^−1^ and 0.003 µM^−1^ s^−1^ for P + 0 and P + 9 complexes, respectively, indicating a 12-fold catalytic preference for ribosomes stalled on a shorter mRNA (Fig. 1d). The rate of ArfB turnover is quite low even for the P + 0 complex, with *k*_cat_ = 0.01 s^−1^. Ribosome recycling factor (RRF) and elongation factor G (EF-G), factors involved in post-termination ribosome recycling^19^, decrease ArfB residence time on the ribosome by up to 30%; release factor 3 (RF3) had very little effect (Fig. 1e).

A ribosome with a long mRNA presents a sense codon in the A site, which is much more likely to be read by a ternary complex (TC) EF-Tu–GTP–aminoacyl-tRNA, which is abundant in the cell, than recruit ArfB, which is expressed in only 0.5 copies per *E. coli* cell, in comparison to over 25,000 copies of RF1^20^. To study the effect of TCs, we measured ArfB activity on ribosome complexes with [^3^H]fMet-tRNA^fMet^ in the P site in the presence of EF-Tu–GTP–[^14^C]Phe-tRNA^Phe^, which is cognate for the second codon, using P + 3 and P + 33 mRNAs (Supplementary Fig. 1a). At high concentration, ArfB is able to impede dipeptide formation on P + 3, but not on P + 33 mRNA (Supplementary Fig. 1b), and, vice versa, ArfB-induced peptide release does not occur on P + 33 complexes in the presence of cognate TC (Supplementary Fig. 1c). ArfB can release fMet from P + 33 only when cognate TCs are completely absent, e.g., in the presence of non-cognate TC alone (EF-Tu–GTP–Val-tRNA^Val^) (Supplemental Fig. 1d).

### Structural mechanism of ribosome rescue by ArfB

**Fig. 2:**
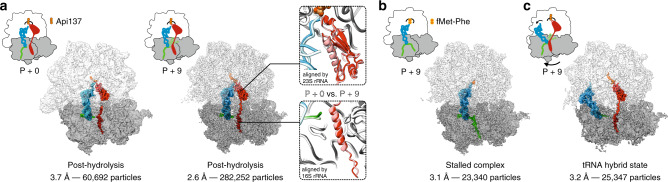
Structural intermediates of ribosome rescue by ArfB as visualized by cryo-EM. For cryo-EM analysis, ArfB was trapped on the ribosome using the antimicrobial peptide Api137. **a** Major states of ArfB-bound P + 0 and P + 9 complexes exhibit a striking structural similarity. Close-ups: Superposition of P + 0 (lighter colors) and P + 9 (darker colors) structures in the PTC (top) and the DC region (bottom). **b** Stalled P + 9 complex with dipeptidyl-tRNA prior to ArfB binding. **c** Post-hydrolysis P + 9 complex with P/E hybrid tRNA. Note the lack of density for mRNA in the mRNA entry channel in the ArfB-bound P + 9 states indicating a highly flexible 3′ extension of mRNA versus the well-defined 3′ extension in the vacant P + 9 complex.

In order to understand how longer mRNAs affect ArfB-mediated rescue, we determined the cryo-EM structures of ArfB bound to P + 0 and P + 9 *E.* *coli* ribosome complexes (Fig. 2, Supplementary Fig. 2 and Supplementary Table 1). To stabilize ArfB on the ribosome, we used the antimicrobial peptide apidaecin-137 (Api137), which is known to trap canonical RFs on the ribosome^21,22^. Similarly to RFs, Api137 stalls ArfB after one round of peptide hydrolysis (Supplementary Fig. 2a). The cryo-EM particle images were sorted in silico for ArfB occupancy and ribosome conformation yielding cryo-EM maps for the major states of P + 0 and P + 9 complexes at 3.7 Å and 2.6 Å resolution, respectively (Supplementary Fig. 2c–e). The two structures are virtually identical within the error of resolution and depict ArfB bound to the ribosome in the post-hydrolysis state with Api137 and deacylated tRNA^Phe^ in the P site (Fig. 2a). ArfB extends from the mRNA entry channel of the 30S subunit to the peptidyl transferase center on the 50S subunit. The N-terminal domain is bound to the A site of the 50S subunit, whereas the C-terminal domain of ArfB folds into an α-helix that occupies the A site and mRNA entry channel on the 30S subunit. The absence of mRNA density past the P-site codon in the P + 9 state suggests that ArfB has displaced the mRNA 3′ extension from the mRNA entry channel. The mRNA is intact under these conditions (Supplementary Fig. 2b), indicating that the A site overhang must have become disordered to allow accommodation of ArfB into the mRNA entry channel.

In the PTC, Gln28 in the universally conserved catalytic GGQ motif of ArfB retains a conformation poised for peptidyl-tRNA hydrolysis, which is stabilized by interaction with Api137 (Supplementary Fig. 2c). The structural details of stalling by Api137 are very similar for ArfB and the canonical RFs^21,22^ (Supplementary Fig. 2c). In both cases, Api137 mimics a nascent peptide chain in the post-hydrolysis state. Api137’s C-terminal hydroxyl group interacts with the ribose hydroxyls at position A76 of the P-site tRNA, thereby stabilizing the tRNA in the PTC. The penultimate Arg17 of Api137 traps the GGQ motif in an active conformation via the guanidinium group that interacts with the side chain carbonyl group of Gln28.

The higher number of cryo-EM particles acquired for the P + 9 complex allowed us to visualize additional intermediates of ribosome rescue: (1) a 3.1 Å map of the pre-hydrolysis complex prior to ArfB binding, with fMetPhe-tRNA^Phe^ in the P site, no Api137, and a clear density for the 9-nucleotide 3′ extension of the mRNA occupying the mRNA entry channel; and (2) a 3.2 Å map of a minor fraction of ribosomes in a post-hydrolysis state with ArfB and Api137 bound and deacylated tRNA^Phe^ in the hybrid P/E state (Fig. 2b and Supplementary Fig. 2e). Because the post-hydrolysis state is structurally very similar with P + 0 and P + 9 mRNAs, in the following we generally refer to the 2.6 Å cryo-EM structure of the P + 9 complex as the major P/P post-hydrolysis state.

### Essential ArfB–ribosome interactions

**Fig. 3:**
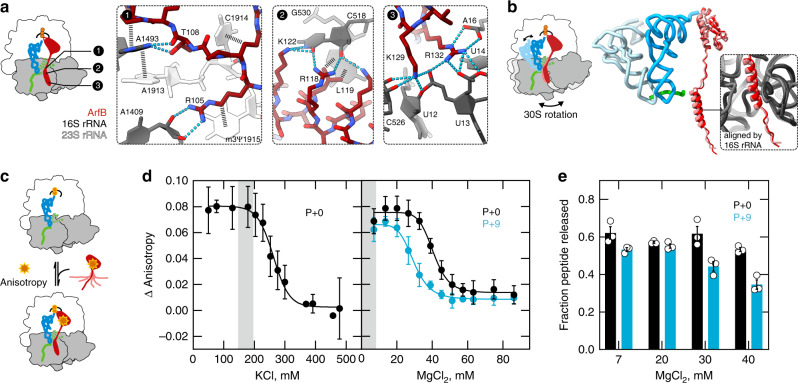
Specific interactions with the ribosome stabilize ArfB in the active state. **a** ArfB residues known to be essential for ribosome rescue^10^ form a network of interactions with the ribosome as seen in the P + 9 cryo-EM structure. Panels 1, 2, 3 depict distinct regions in ArfB indicated in the schematic (left). **b** Subunit rotation changes the position, but not the interactions of ArfB on the ribosome in the P + 9 hybrid state cryo-EM structure. **c** Experimental assay to measure the affinity of ArfB binding to stalled ribosomes using the anisotropy change of fluorescein-labeled ArfB_GAQ_ (ArfB_GAQ_(Flu)). **d** Role of electrostatic interactions in ArfB binding to the ribosome. P + 0 or P + 9 complexes (0.15 µM) with ArfB(Flu) (0.05 µM) at different KCl (left) or MgCl_2_ (right) concentrations. IC_50_ is ~260 mM for KCl and ~40 mM for MgCl_2_ (P + 0) or ~30 mM for MgCl_2_ (P + 9). The range of physiological ion concentrations is highlighted in gray (180–200 mM for K^+^ and 2–3 mM for Mg^2+^; Ref. ^23^). Error bars indicate the SEM of three biological replicates, and the solid lines are dose-response fits. **e** Effect of Mg^2+^ concentration on ArfB activity. P + 0 and P + 9 complexes (0.1 µM) were incubated with ArfB (1 µM) for 5 min at 20 °C. Error bars indicate the SEM of three biological replicates.

The present 2.6 Å cryo-EM map shows how ArfB interacts with the ribosome in the post-hydrolysis complex. The density for ArfB, in particular in the C-terminal domain, is significantly better in the cryo-EM structure than in the previous crystal structure^15^, which allowed us to improve model accuracy, including register shifts in the N- and C-terminal domains (Supplementary Fig. 3a). In agreement with mutational data, the cryo-EM-based model shows that the essential residues Arg105, Arg118, Leu119, Lys122, Lys129, and Arg132 form intricate side chain contacts with the ribosomal RNA (rRNA), whereas most nonessential side chains that are not involved in any interactions are less well-resolved (Fig. 3a and Supplementary Fig. 3b). The essential positively charged amino acids interact via their guanidinium and ε-amino groups with negatively charged groups of 16S rRNA (and 23S rRNA for Arg105), whereas the hydrophobic Leu119 stacks with its isobutyl group onto the guanine of G530. Moreover, the key functional residues, Lys129 and Arg132, also show the most intricate interactions. For instance, the side chain of Arg132 alone forms six non-covalent interactions; mutating Arg132 even to a Lys completely abolishes catalysis, whereas the binding to the ribosome is reduced by twofold only^10^. Notably, the network of ArfB–16S rRNA interactions that we observe in the major P/P post-hydrolysis state appears to be very stable and persists upon 30S subunit rotation, as evident from the comparison with the minor P/E post-hydrolysis state (Fig. 3b).

To further understand the nature of the ArfB–ribosome interactions and probe them not only in the post-hydrolysis state revealed by the cryo-EM, but also in the pre-hydrolysis state, we studied the effect of monovalent (KCl) and divalent (MgCl_2_) ions on ArfB binding to P + 0 and P + 9 complexes (Fig. 3c, d). To capture the factor in the pre-hydrolysis state, we used ArfB_GAQ_(Flu), a hydrolysis-deficient ArfB mutant (Gly27 to Ala), with a fluorescein attached at position 96 (Supplementary Fig. 4a–c). Binding to the ribosome is monitored as an increase of the fluorescein anisotropy. The binding is salt sensitive, indicating that electrostatic interactions play an important role (Fig. 3d). However, at ionic strength within the physiological range^23^ the binding of ArfB is not perturbed, as the inhibitory concentration IC_50_ for the P + 0 complex is ~260 mM for KCl and ~40 mM for MgCl_2_. Thus, ArfB binding is not mediated by nonspecific electrostatic interactions, but rather by strong side chain-specific interactions such as those we observe in the cryo-EM structure. For the P + 9 complex, the tendency is similar, but the IC_50_ value for Mg^2+^ is somewhat lower (~30 mM) than for the P + 0 complex, indicating a difference in binding stabilities (Fig. 3d). We also measured the effect of Mg^2+^ on peptidyl-tRNA hydrolysis (Fig. 3e). At high ArfB concentrations, hydrolysis is not affected by Mg^2+^ demonstrating that Mg^2+^ ions do not affect the chemistry step itself. Taken together, our cryo-EM data and binding measurements, as well as the published mutagenesis data^10^, strongly suggest that the essential C-terminal residues of ArfB play a major role in specifically stabilizing the catalytically active state of the ArfB–ribosome complex.

### Kinetics of ArfB initial binding followed by engagement

**Fig. 4:**
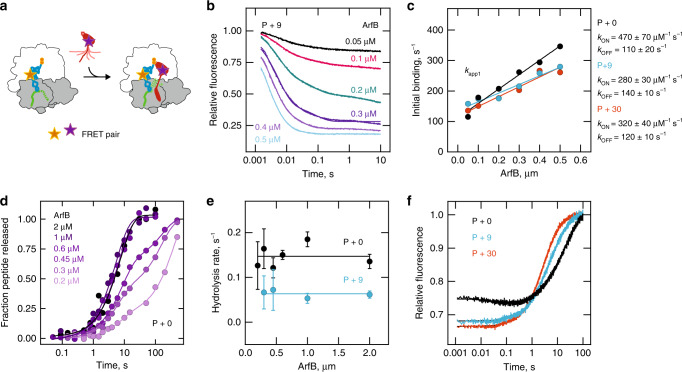
A dynamic model of ArfB recruitment. **a** Experimental assay to monitor binding of ArfB to the ribosome in real time. P + *n* ribosome complexes containing fluorescein-labeled fMetPhe-tRNA^Phe^ (P + *n*(Flu)) are mixed with quencher-labeled ArfB (ArfB(540Q)) and fluorescein quenching is monitored in real time in a stopped-flow apparatus. **b** Time courses of ArfB binding with fixed concentration of P + 9(Flu) (0.015 µM) and increasing concentrations of ArfB(540Q) (0.05-0.5 µM) (20 °C). Lines indicate three-exponential fits. **c** Kinetics of initial binding of ArfB(540Q) to P + 0(Flu), P + 9(Flu), and P + 30(Flu) complexes. Plotted is the concentration dependence of the apparent rate constant (*k*_app1_) for the predominant association phase in (**b**). Data represents the mean values of two biological replicates with up to six technical replicates each. The association (*k*_ON_) and dissociation (*k*_OFF_) rate constants are determined by linear fitting of the concentration dependence. Errors of k_ON_ and k_OFF_ values are SEM of the fit. **d** Kinetics of peptidyl-tRNA hydrolysis. Time courses of hydrolysis with P + 0 ribosome complex (0.15 µM) and ArfB (0.2–2 µM) (37 °C). Data represented as mean values of two biological replicates. **e** Comparison of peptidyl-tRNA hydrolysis rates on P + 0 and P + 9 complexes. Values are obtained by exponential fitting of the rapid phase of the hydrolysis time courses (**d** and Supplementary Fig. 4f). Error bars represent the SEM of the fit. **f** Dissociation of ArfB from P + 0, P + 9, and P + 30 complexes. The release of ArfB_GAQ_(540Q) (0.1 µM) from the pre-hydrolysis complexes P + 0(Flu), P + 9(Flu), and P + 30(Flu) (0.015 µM) was initiated by rapid mixing with unlabeled P + 0 complexes (1 µM) (20 °C).

Because ArfB attains very similar conformations on P + 0 and on P + 9 post-hydrolysis complexes, we sought to identify a potential mRNA discrimination step prior to hydrolysis. First, we monitored ArfB binding to the ribosome by fluorescence resonance energy transfer (FRET) using a donor–acceptor pair with a fluorescein-labeled tRNA in the P site and a fluorescence quencher-labeled ArfB (ArfB(540Q)) (Fig. 4a); the respective ribosome complexes are denoted P + 0(Flu), P + 9(Flu), and P + 30(Flu). The activity of the labeled components is not affected by fluorescence labeling (Supplementary Fig. 4d). The experiments were carried out at 20 °C, because at 37 °C the binding was too fast to measure by the stopped-flow technique.

Binding of ArfB(540Q) to P + 0(Flu) (Supplementary Fig. 4e), P + 9(Flu) (Fig. 4b) or P + 30(Flu) results in fluorescence quenching due to proximity of the quencher to the fluorophore. To determine the association and dissociation rate constants, we measured the kinetics of binding at increasing concentrations of ArfB(540Q) in excess over a constant concentration of labeled ribosome complexes. Exponential fitting of the resulting time courses yields three distinct apparent rate constants, indicative of three binding phases (Fig. 4c and Supplementary Fig. 4e, f). With P + 0, P + 9, or P + 30 complexes, the predominant phase contributing up to 80% of the observed FRET change is very rapid; the two slower phases together contribute about 20% of the FRET change (Fig. 4c, Supplementary Fig. 4e, f and Supplementary Table 2). In a simplest model, if the rapid phase reports on the association of ArfB to the ribosome, and the slower phases represent subsequent rearrangement steps, the concentration dependence of the apparent rate constants is expected to be linear for the rapid phase and hyperbolic for the slower phases. Surprisingly, this turned out not to be the case; rather, all three apparent rate constants increased linearly with ArfB concentration (Fig. 4c and Supplementary Fig. 4f), indicating that all phases reflect binding, albeit apparently for different ArfB populations. We then estimated the rate constants of binding (*k*_ON_) and dissociation (*k*_OFF_) for the predominant rapid phase, which represent the values for the majority of ArfB molecules (Fig. 4c). The binding is very rapid with *k*_ON_ of ~500 µM^−1^ s^−1^ for P + 0 and ~300 µM^−1 ^s^−1^ for P + 9 and P + 30. The initial binding complex is labile, with *k*_OFF_ of ~110 s^−1^, ~140 s^−1^, and ~120 s^−1^ for P + 0, P + 9, and P + 30, respectively, which shows that the initial binding is independent of the mRNA length. The high values of the *k*_ON_ and *k*_OFF_ independent of the mRNA length suggest a scanning step where ArfB rapidly binds to and can dissociate from ribosomes regardless of whether the mRNA entry channel is occupied or not.

To understand the nature of the rapid association step, we tested the effect of Mg^2+^ concentrations (Supplementary Fig. 4g). We have chosen Mg^2+^ rather than monovalent cations, because Mg^2+^ disrupts rapid nonspecific binding of initiation factor 3 (IF3) to the ribosome^24^; binding of ArfB to the ribosome may be driven by similar interactions. However, ArfB association with the ribosome is not affected by high ion concentrations (Supplementary Fig. 4h), which suggests that the initial binding is not driven by electrostatic interactions.

Given that the initial binding is insensitive to the mRNA length, a subsequent step must account for the observed inhibition of peptidyl-tRNA hydrolysis with the long mRNAs (Fig. 1c). In a simplest model, the rate of the chemistry step itself may depend on the mRNA length, i.e., due to misalignment in the transition state. At saturating concentrations of ArfB, the rate of hydrolysis is ~0.15 s^−1^ on P + 0 complexes, and 0.06 s^−1^ on P + 9 complexes (Fig. 4d, e and Supplementary Fig. 4i). If the measured rate reflected the chemistry step, it is expected to be pH-dependent, as previous experiments on the kinetics of peptidyl-tRNA hydrolysis by RF1 and RF2 revealed a strong pH dependence of the reaction^25,26^. However, for the ArfB-catalyzed reaction, the hydrolysis rate is identical at pH 6.8, 7.4, and 8.0 for both P + 0 and P + 9 complexes (Supplementary Fig. 4j). This strongly suggests that hydrolysis is rate-limited by a step after initial binding, but preceding the chemistry step. The rate of this additional step, which we call an engagement step, depends on the mRNA length (Fig. 4e), whereas the subsequent chemistry step itself must be faster than engagement.

To measure the stability of ArfB binding to stalled ribosomes prior to peptidyl-tRNA hydrolysis we used ArfB_GAQ_ and a FRET pair described above (Fig. 4f and Supplementary Fig. 4b). We formed complexes of ArfB_GAQ_(540Q) with P + 0(Flu), P + 9(Flu), or P + 30(Flu), and then rapidly mixed them with a 10-fold excess of unlabeled P + 0 complexes. Fluorescence increase over time reports on the dissociation of ArfB_GAQ_(540Q) from the ribosome (Fig. 4f). Exponential fitting of the time courses (Methods) required a minimum of two dissociation steps, a slow one with a rate constant of 0.04 s^−1^ (P + 0), 0.07 s^−1^ (P + 9) or 0.1 s^−1^ (P + 30) and a faster one of about 0.4 s^−1^, similar for the three complexes (Supplementary Table 3). The difference is in the fraction of the slow vs. fast steps, with about 80% of P + 0 compared to 50–40% of P + 9 and P + 30 complexes dissociating slowly, as indicated by the proportion of the fluorescence change amplitude for each step. The slowly dissociating population likely represents the complexes captured by cryo-EM; hence the similar conformation of P + 0 and P + 9. The population of complexes that dissociate more rapidly may represent either a step before the full engagement of ArfB, i.e., when not all interactions of ArfB with the ribosome are formed, or a heterogeneity within the ribosome complexes, with a larger proportion of rapidly dissociating complexes when the mRNA is long. As we cannot distinguish between these two possibilities, we report average dissociation rate constants of 0.06 s^−1^, 0.23 s^−1^, and 0.33 s^−1^ for P + 0, P + 9, and P + 30, respectively, which is a characteristic of the ensemble as a whole. In summary, our rapid kinetics analysis identifies an engagement step prior to peptidyl-tRNA hydrolysis that results in a selective stabilization and more rapid rescue reaction for those complexes that have a short truncated mRNA.

## Discussion

Our rapid kinetics analysis shows that ribosomes stalled on short truncated mRNAs are the physiologically relevant substrate for ArfB-dependent rescue. The residual ArfB activity is substantially reduced when the mRNA exceeds the P-site codon by 9 nt or more. When the mRNA is long, ArfB is unable to compete with cognate TCs and can rescue the ribosomes only if a cognate TC is absent. The latter is, however, not a realistic scenario in vivo, because in bacteria the codon frequency correlates with aa-tRNA abundance, i.e., some aa-tRNA are rare but not missing. Even if aa-tRNA pools are exhausted, e.g., by starvation, the concentrations of rare tRNAs isoacceptors are less responsive to starvation conditions than those of abundant tRNAs^27^. Thus, at cellular concentrations, ArfB activity is too low to interfere with on-going translation of long mRNAs. We therefore conclude that a role for ArfB in rescuing ribosomes that pause at a rare codon cluster is unlikely; rather, ArfB acts on complexes containing an mRNA truncated just past the P site.

In the context of ribosome rescue in *E. coli*, the specific role of ArfB remains unclear, as ArfB has the same preference for ribosomes stalled on short mRNAs as tmRNA-SmpB and ArfA^11–13^. As suggested earlier, ArfB may be simply not as crucial in *E. coli* as in other species that lack one or both of the other rescue systems^5^. Notably, its human homolog ICT1 is the only rescue factor that is found in eukaryotic mitochondria^8^. The exact function of ICT1 is unclear, because it is also found as an integral protein of the large ribosomal subunit, mL62^28,29^. The activity of ICT1 as a peptidyl-tRNA hydrolase is essential for cell viability^2,3^, and some studies have proposed that ICT1 catalyzes termination on mitochondrial transcripts with non-canonical stop codons AGA and AGG^30,31^. Assuming that ArfB and ICT1 have a similar preference for mRNA with short overhangs, a role for termination seems unlikely, as the mRNAs in question extend 14 nt past the A site^32,33^, a length at which ArfB activity is drastically reduced. On the other hand, mitochondrial mRNAs are polyadenylated, with polyadenylation playing transcript-dependent roles in regulating protein synthesis^34,35^. The sensitivity to mRNA length could create interesting opportunities for regulation of translation and mRNA stability in mitochondria by ICT1.

**Fig. 5:**
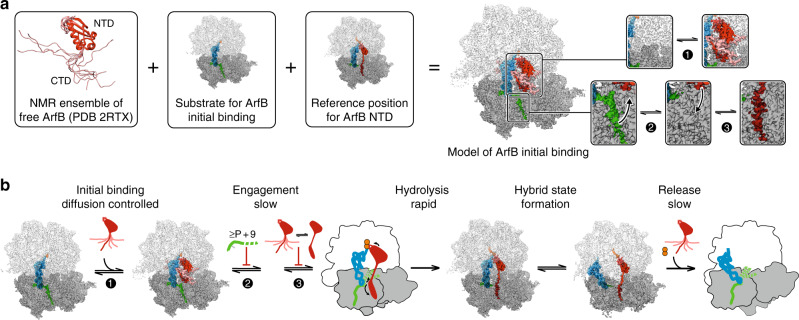
Mechanism of ribosome rescue by ArfB based on rapid kinetics and cryo-EM data. **a** Model for initial binding of ArfB, based on the NMR ensemble of free ArfB^10^ and cryo-EM structures of the P + 9 ribosome-ArfB complex. 1, 2, 3 illustrate the key steps of the mechanism. **b** Mechanism of ArfB action. When the ribosome stalls on a truncated mRNA, ArfB can rapidly bind regardless of the mRNA length (1). Subsequent conformational rearrangements allow the factor to probe the mRNA entry channel; if there is mRNA extending past the P site, the mRNA must first move out of the mRNA entry channel (2), a process that occurs more slowly with longer mRNAs. The binding and folding of the ArfB C-terminal domain result in the engagement of ArfB on the 30S subunit (3), which allows the rapid hydrolysis reaction to occur via the GGQ motif in the PTC, followed by peptide release, ribosome rotation and movement of the tRNA into the hybrid state, and ArfB dissociation.

Our biochemical and structural data reveal the mechanism of ArfB-mediated ribosome rescue. In combination with the reported solution structures of free ArfB, we propose a structural model explaining the rapid mRNA-independent initial binding and subsequent mRNA-dependent engagement step of ArfB. Structures of free ArfB^10^ and of its human ortholog ICT1^9^ determined by NMR indicate that in solution the linker and C-terminal domains of the two factors are largely disordered, whereas the structures of their catalytic N-terminal domains are similar to that in the ribosome-bound state and only the catalytic GGQ motif itself is disordered. The structure of the linker determines the relative orientation of the N- and C-terminal domains, indicating that in solution the molecule is highly dynamic due to the linker flexibility. To understand how this heterogeneous ensemble of conformers binds to the ribosome, in particular with an mRNA occupying the entry channel, we docked the structures of the NMR ensemble of free ArfB onto the P + 9 complex using the N-terminal domain for orientation (Fig. 5a). The resulting model provides a simple explanation for the kinetic data (Fig. 5b). If an ArfB molecule initially docks onto the ribosome through the contact between the ArfB N-terminal domain and the 50S subunit, the interdomain linker would be flexible enough to accommodate the C-terminal domain into the intersubunit space. The positively charged C-terminal tail may form unspecific interactions with the negatively charged rRNA backbone at this stage. Most importantly, such initial docking does not require the mRNA to move out of the entry channel, explaining why the rapid initial binding of ArfB is independent of the mRNA length. The theoretical encounter frequency^36^ for ArfB and the ribosome is ~9 × 10^9^ M^−1 ^s^−1^, whereas the *k*_ON_ for the major fraction of ArfB molecules is 5 × 10^8^ µM^−1 ^s^−1^. This difference may suggest that for a fraction of ArfB molecules (~10%) arriving in an optimal orientation, the reaction is diffusion controlled, whereas the majority of molecules require several attempts before docking, which slows down the effective association. The remaining small fraction of ArfB molecules that bind even slower (Supplementary Fig. 4f) may arise from those ArfB conformations where the C-terminal domain clashes with the ribosome and is too slow to rearrange. At this step, no catalytic activation takes place in agreement with the disordered GGQ motif in the NMR structures.

In the subsequent engagement step, docking of the C-terminal tail into the mRNA channel requires movement of the A-site mRNA extension out of the channel, consistent with the notion that the engagement is substantially slower for a longer mRNA. However the rate of engagement is also low with the P + 0 mRNA, which may be due to the slow ordering and accommodation of the ArfB C-terminal domain into the mRNA channel. The favorable enthalpic contribution of forming specific interactions with the ribosome may be offset by the entropic cost of folding of the C-terminal domain. In contrast to the initial binding, the engagement step is salt sensitive, suggesting an important role of electrostatic interactions. The pliability of the flexible linker region would allow the basic C-terminal domain to fit into the intersubunit space, possibly guided by electrostatic steering by the acidic phosphate backbone of the ribosome. ArfB C-terminal domain forms a network of hydrogen bonds and stacking interactions with key residues in the decoding center of the ribosome. Many of these interactions are electrostatic in nature and very stable at physiological salt concentrations. Once ArfB has been fully accommodated into the mRNA channel, catalysis is rapid, in agreement with the similar structures for the P + 0 and P + 9 post-hydrolysis states. Previous work has shown that the N-terminal domain on its own cannot hydrolyze peptidyl-tRNA^10^. This is consistent with the key role of the ArfB interdomain linker in activating its hydrolytic activity; how exactly the folding and binding of the C-terminal domain activates peptidyl-tRNA hydrolysis more than 40 Å away remains unknown.

Our kinetic and structural data explain why ArfB is more active when the mRNA extension is short. The steady-state kinetic data indicate that the specificity of ArfB binding (*k*_cat_/*K*_M_) is decreased by >10-fold when the mRNA extension past the P-site codon exceeds 9 nt. The lower catalytic activity of ArfB is due to the slower engagement step and to a smaller fraction of molecules that are stabilized in the catalytically active conformation. Overall, ArfB follows the same strategy to select for the rescue substrates as ArfA–RF2^37–41^: they activate peptide release only after the mRNA-sensing domain is accommodated in the mRNA channel on the 30S subunit.

After peptidyl-tRNA hydrolysis and peptide release, the ArfB–ribosome complex adopts a rotated conformation with tRNA in a hybrid state. The flexible linker domain of ArfB allows both ArfB domains to retain their interactions with the 50S and 30S subunits. This may explain why turnover of ArfB is slow compared to other factors interacting with the ribosome: while rotation of the ribosomal subunits destabilizes RF1 and RF2 binding to the ribosome^42^, ArfB retains its tight interactions in the non-rotated and rotated state. The *k*_cat_ value of 0.01 s^−1^ is much lower than the single-round hydrolysis rate of 0.15 s^−1^ for the P + 0 complex, suggesting that for short truncated mRNAs the overall ArfB cycle is limited by ArfB dissociation. The tight interactions that persist after peptide hydrolysis likely contribute to the slow dissociation rate, which may encompass the rates for unbinding as well as unfolding of the ArfB C-terminal domain. With the longer mRNA constructs, where the rate of hydrolysis is low, engagement of ArfB might become the rate-limiting step. The presence of recycling factors RRF and EF-G may accelerate turnover by precluding ArfB from unproductively binding to post-termination ribosomes. Whether additional factors are required to disengage the tight binding of ArfB to the ribosome after peptide release is unclear at present.

In conclusion, our studies show how protein dynamics contributes to molecular recognition and discrimination in a large ribonucleoprotein complex. The disordered linker region of ArfB helps the factor to rapidly dock and scan the state of the mRNA on the ribosome, and to select the right substrate. The linker also guides the C-terminal domain into its confined binding pocket in the mRNA entry channel and helps the factor to adjust to different ribosome conformations. Binding of the C-terminal domain is stabilized by specific electrostatic interactions that define the shape of ArfB in the complex and ultimately lead to catalytic activation of the GGQ motif in the N-terminal domain. Thus, flexibility allows ArfB to bind rapidly and non-specifically to its target first, followed by ordering of the C-terminal domain and formation of tight specific interactions, as has been suggested for dynamic, intrinsically disordered proteins^18,43^.

## Methods

### Reconstitution of stalled ribosome complexes

All experiments were carried out in HKM_7_ buffer (50 mM HEPES pH 7.4, 30 mM KCl, 7 mM MgCl_2_) unless otherwise stated. 70S ribosomes from *E. coli* MRE600, IF1, IF2, IF3, EF-Tu, EF-G, RRF, f[^3^H]Met-tRNA^fMet^, [^14^C]Phe-tRNA^Phe^, and [^14^C]Phe-tRNA^Phe^(Flu) were prepared using published protocols^44–46^. [^14^C]Phe-tRNA^Phe^(Flu) is labeled with fluorescein at thioU8. Truncated mRNAs of varying length were in vitro transcribed using T7 RNA-polymerase from a T7 promoter on a DNA template derived from the *E. coli lepB* gene with the second codon mutated to encode phenylalanine, then purified over a HiTrap Q HP column.

Stalled ribosome complexes were prepared by translating the first two codons on truncated mRNAs using published protocols^47,48^. Briefly, 70S ribosomes were mixed with 1.5-fold excess of initiation factors, 3-fold excess of f[^3^H]Met-tRNA^fMet^, and 3-fold excess of mRNA in HAKM_7_ buffer (HKM_7_ buffer supplemented with 70 mM NH_4_Cl) containing 1 mM dithiothreitol (DTT) and 1 mM GTP, and incubated for 45 min at 37 °C. Initiation efficiency, determined by nitrocellulose filtration and scintillation counting using QuantaSmart (Perkin Elmer), was >90% for all mRNAs. Ternary complexes were formed with 3-fold excess of [^14^C]Phe-tRNA^Phe^ or [^14^C]Phe-tRNA^Phe^(Flu) over ribosomes and 1.5 µM EF-G, and a 2-fold excess of EF-Tu incubated with 1 mM GTP, 3 mM phosphoenolpyruvate (PEP), and 0.5 mg mL^−1^ pyruvate kinase in HAKM_7_ buffer for 15 min at 37 °C. To form P + 0 complexes, initiation complexes (IC) were mixed with TC for 2 min at room temperature to translate the second codon. To form P + *n* complexes (*n* = 3, 6, 9, 12, 15, 24, 30, and 99), the ribosomes were supplied with the same TC so that the ribosome is stalled with the second codon of the mRNA in the P site. The resulting complexes were purified by ultracentrifugation over a cushion of 1.1M sucrose in HAKM_20_ buffer (HAKM_7_ buffer containing 20 mM MgCl_2_). The pellets were resuspended in HKM_7_ buffer and quantified by liquid-liquid scintillation counting, then flash frozen and stored at −80 °C. Dipeptide formation under these conditions was at least 80%.

### ArfB purification

The sequence of the *yaeJ* gene encoding ArfB was cloned from *E. coli* K12 strain into a vector carrying an N terminal 6xHis tag followed by a SUMO tag. Overexpression and cell lysis were carried out using a published protocol^15^. Tagged ArfB was isolated from the cell lysate by incubation with Ni-IDA beads in buffer A (40 mM HEPES pH 7, 300 mM KCl, 7 mM MgCl_2_, and 10 mM β-mercaptoethanol) for 1 h at 4 °C. Following elution with buffer A supplemented with 600 mM imidazole, fractions containing ArfB were dialyzed into buffer A overnight at 4 °C in the presence of Ulp1 protease to cleave the SUMO tag. Untagged ArfB was further purified via cation-exchange chromatography over a HiTrap HP SP column. ArfB concentration was determined by comparing SDS-PAGE band intensities to a standard curve generated by known amounts of IF3 that was run on the same gel. For fluorescence-labeled ArfB, amino acid residues at positions 96 and 112 were changed to cysteine, and labeled with a thiol-reactive fluorescein or ATTO540Q using established protocols^46^. Free dye was removed using a PD MidiTrap G-25 column (GE Healthcare). Labeling efficiency, estimated by absorbance measurements of the dye and SDS-PAGE based estimation of protein concentration, was ~60%.

### Hydrolysis assays

Single-round peptidyl-tRNA hydrolysis was monitored by rapidly mixing P + 0 complexes and ArfB in a quenched-flow apparatus at 37 °C. Released peptides were quantified using a published protocol^21^: at each time point, the reaction was stopped by adding a quenching solution of chilled 10% trichloroacetic acid (TCA) and 50% ethanol. The samples were kept on ice for 30 min, then the precipitated ribosomes and peptidyl-tRNA were spun down for 15 min at 16,000 × *g* at 4 °C. The released peptides in the supernatant were quantified by liquid-liquid scintillation counting. Time courses were evaluated by exponential fitting in GraphPad Prism using one or two-exponential terms, depending on the ArfB concentration used. For high ArfB concentrations, one exponential fitting was sufficient. At low ArfB compared to the ribosome concentration (Fig. 4d), the first reaction round was successful only on a fraction of ribosomes and the completion of the reaction required multiple rounds of ArfB binding, which results in two-exponential behavior; of the two apparent rate constants, the faster represents the single-round rate constant.

Multiple-turnover peptidyl-tRNA hydrolysis was measured at 37 °C by incubating ArfB with at least 10-fold excess of stalled ribosome complex. At each time point, an aliquot of the mixture was quenched as described above. Initial velocity of the reaction was calculated as the slope of the linear fit of the time course, after subtracting background peptide drop-off, as measured in a parallel reaction without ArfB. Michaelis–Menten constants *k*_cat_, *K*_M_, and *k*_cat_/*K*_M_ were calculated from the hyperbolic fit of initial velocity plotted against substrate concentration. To assess the effect of translation factors on ArfB turnover, the HKM_7_ buffer was supplemented with 3 mM PEP, 1 mM GTP, and 1 mg mL^−1^ pyruvate kinase. Initial velocities of the hydrolysis reaction were measured in the presence of RF3, RRF, and EF-G.

### ArfB activity assay in the presence of elongation factors

To observe the interplay between ArfB and ongoing translation, IC termed P + 3 and P + 33 were prepared as described above, albeit without the addition of TC. ArfB (0.3 or 2 µM) was mixed with IC (0.5 µM) and TC (0.25 µM EF-Tu, 1 µM [^14^C]Phe-tRNA^Phe^, 1 mg mL^−1^ pyruvate kinase, 3 mM PEP, and 1 mM GTP) and incubated at 37 °C for 120 s. Each reaction was run twice, with one sample quenched with 0.1× sample volume 5M potassium hydroxide (KOH) then hydrolyzed for 30 min at 37 °C, and subsequently used to quantify the amount of dipeptides formed. The other sample was quenched with 500 µL 10% TCA and 50% ethanol, and processed to quantify the amount of [^3^H]fMet released.

Dipeptides were quantified using a published protocol^50^. Briefly, the samples quenched with KOH were neutralized with acetic acid and analyzed by reversed phase HPLC (Chromolith RP8 100–4.6 mm column, Merck), over a 0–65% acetonitrile gradient in 0.1% TFA. Fractions were analyzed by scintillation counting, with the fractions containing both ^3^H and ^14^C counts identified as dipeptide-containing fractions.

Hydrolysis in the presence of non-cognate TC was measured at 37 °C. Non-cognate TC was formed for 15 min at 37 °C with 10 µM EF-Tu, 5 µM Val-tRNA^Val^, 0.5 mg mL^−1^ pyruvate kinase, 1.5 mM PEP, and 0.5 mM GTP (all concentrations reflect final concentrations in the experiment). TC was mixed with an equal volume of purified P + 3 or P + 33 in the presence of 0.1 µM ArfB and 2 µM ArfB, respectively. To assess the effect of translocation on hydrolysis, 2 µM EF-G was also added to a set of samples. Aliquots were taken at time points up to 5 min, then processed to quantify the released peptides.

### Pre-steady-state binding assays

All stopped-flow experiments were performed at 20 °C, with an excitation wavelength of 465 nm and a KV500 cutoff filter (Schott), and using an SX-20MV stopped-flow machine (Applied Photophysics). Fluorescence data was collected using Pro-Data SX (Applied Photophysics). For the binding reaction, equal volumes of ArfB(540Q) labeled at position 96 and P + 0(Flu) complex were rapidly mixed to a final concentration of 0.05, 0.1, 0.2, 0.3, 0.4, and 0.5 µM ArfB, and 0.015 µM P + 0(Flu) complex. Fluorescence quenching was recorded over time. For the pre-hydrolysis dissociation reaction, 0.2 µM ArfB_GAQ_(540Q) was incubated with 0.03 µM P + 0(Flu) for 1 min at room temperature to form the complex, then rapidly mixed with an equal volume of 2 µM unlabeled P + 0 complex. Recovery of fluorescence following dissociation of the quencher-labeled ArfB was recorded over time. The resulting time courses were fit with exponential equations using GraphPad Prism. Average dissociation rate constants were calculated by summation of the product of the apparent rate and the amplitude of each exponent. All fluorescence traces were then normalized by the highest level of fluorescence as extrapolated by the fit.

While the rates are clearly different for the P + 0 and P + 9 or P + 30 complexes, we note that for technical reasons the dissociation rate constants were measured at 20 °C, whereas the single- and multiple-turnover hydrolysis rates are measured at physiological temperature. Thus, the measured dissociation rates provide a lower limit to the values expected in vivo. Also the precise value for the chemistry step is not known, as it is rate-limited by the preceding engagement step. These uncertainties prevent us from calculating the selectivity of ArfB from the elemental rate constants, as we previously have done for tRNA selection^49^.

### Equilibrium binding assays

All binding experiments were carried out at 20 °C. To assess the effect of salt concentration on ArfB binding, ArfB[Flu] was allowed to bind to P + 0 complexes in HK_50_M_7_ buffer (HKM_7_ buffer with 50 mM KCl). Then, KCl was titrated into the sample, and the anisotropy value at each KCl concentration was recorded. A titration of the unbound protein was performed in parallel and the anisotropy values of about 0.2 subtracted; to account for light scattering, polarized light intensities of P + 0 complexes at each KCl concentration were also subtracted. The resulting curve was fit with a log(inhibitor) dose-response (variable slope) equation. Anisotropy was recorded using the FluorEssence software (Horiba).

The integrity of the ribosome-bound mRNA following ArfB binding was assayed using ribosome-nascent chain complexes labeled at the 3′ end of mRNA with fluorescein (provided by Bee-Zen Peng, MPIbpc). We expect that if the mRNA was cleaved in the presence of ArfB, the anisotropy of the free 3′ mRNA fragment must be significantly lower than that of the ribosome-bound mRNA. The complexes had a model dipeptide (fMetPhe-tRNA^Phe^) and 36 nt of mRNA extending past the P site. Anisotropy of the attached dye was measured before and after incubation of 0.1 µM ArfB with 0.01 µM ribosomal complex.

### Cryo-EM analysis

For cryo-EM of ArfB-bound P + 0 complexes, P + 0 complexes (purified as described above) were diluted to 0.24 µM with HKM_7_ + DDM-Buffer [50 mM Hepes pH 7.4, 30 mM KCl, 7 mM MgCl_2_, 0.1 % w/v Dodecyl-β-D-maltosid (DDM)]; ArfB was diluted to 3 µM using the same buffer. In total, 2 µL of ribosome complex was supplemented with 0.4 µL ArfB and 0.33 µL Api137 (0.6 mM in 50 mM Hepes pH 7.2, 100 mM potassium acetate, 25 mM magnesium acetate; synthesized by NovoPro Biosciences Inc.) and filled up to 4 µL with HKM_7_ + DDM-Buffer leading to final concentrations of 0.12 µM of P + 0 complex, 0.30 µM ArfB (2.5-fold excess) and 50 µM Api137. 3 µl of the sample were applied to glow discharged 2 nm precoated Quantifoil R3/3 holey carbon support grids. Grids were blotted for 2–3 s and vitrified using the Vitrobot Mark IV (ThermoFisher Eindhoven). Data collection was performed in movie mode—10 frames at a dose of 2.5 electrons per Å^−2^ per frame—with a Falcon II direct electron detector (ThermoFisher Eindhoven) on a Titan Krios electron microscope (ThermoFisher Eindhoven) at 300 kV, a pixel size of 1.084 Å and a defocus range from 1.1 to 2.3 µm using the semi-automated software EM-TOOLS (TVIPS GmbH).

For cryo-EM of ArfB-bound P + 9 complexes, traces of sucrose were removed from P + 9 complexes (purified as described above) using Zeba Spin Desalting Columns (7K MWCO, ThermoFisher). The complexes (0.4 µM) were then incubated with ArfB (1.5 µM) and Api137 (50 µM) for 10 min at 37 °C in buffer B (50 mM HEPES, 30 mM KCl, 7 mM MgCl_2_, pH 7.4). Cryo-EM grids were prepared by applying 5 µl of the resulting complexes onto EM grids (Quantifoil 3.5/1 μm, Jena) covered with pre-floated continuous carbon, manually blotted with filter paper (Whatman #1) and vitrified using a custom-made plunge-freezing device operated at 4 °C and 95% humidity. In total, 4096 × 4096 image movie stacks—40 frames per image, ~50 ± 5 electrons per Å^2^ total electron dose, 0.2–2.5 µm defocus—were acquired in integration mode on a Falcon 3 direct detector (ThermoFisher Eindhoven) at 300 kV acceleration voltage with a Titan Krios G1 microscope (ThermoFisher Eindhoven) equipped with a XFEG electron source (ThermoFisher Eindhoven) and a spherical aberration (Cs)-corrector (CEOS Heidelberg) using the software EPU 2.1 (ThermoFisher Eindhoven) for acquisition and CETCORPLUS 4.6.9 (CEOS Heidelberg) for tuning of the Cs-corrector.

Data processing of images of P + 0 and P + 9 complexes was performed using a similar strategy for both data sets as described in the following. Image movie stacks were motion corrected using the software MCOR2^51^, CTF parameters were estimated using GCTF^52^ and ribosome particle images were selected using GAUTOMATCH (K. Zhang, MRC-LMB, Cambridge). All subsequent cryo-EM image processing was performed using RELION 2.1^53^ and 3.0^54^. The two data sets represented mixtures of different ribosome populations and were therefore sorted computationally in a hierarchical manner (Supplementary Fig. 2d). First, ribosome particle images were sorted according to data quality by 2D classification and 3D classification at 3.252 Å per pixel (P + 0) and 4.64 Å per pixel (P + 9; step 1 and 2). All following steps were performed at the final pixel sizes of 1.07 Å (P + 0) and 1.16 Å (P + 9). The P + 0 data were subsequently classified according to ArfB occupancy by focused classification with signal subtraction (step 3) and by global classification according to ribosome conformation (step 4). The latter step resulted in three populations corresponding to different states of ribosome conformation: (1) the non-ratcheted ground state, (2) an intermediate state of rotation, and (3) a fully rotated ratcheted state. Due to the low particle numbers for the two rotated states, only particles of the major ground state were further processed. These particles were sorted again for presence of ArfB resulting in a homogeneous particle population of ArfB-bound P + 0 ribosome complexes (step 5) that was refined to a final resolution of about 3.7 Å (Supplementary Fig. 2e). For the P + 9 data per-particle-motion correction was performed by RELION’s Bayesian polishing approach in step 6 and the resulting particles were sorted by 3D classification according to global ribosome conformation (step 7). The resulting two populations—one with ribosome particles in the ground state (non-rotated) and the other one with particles showing intersubunit rotation (rotated)—were each further classified by focused classification with signal subtraction according to tRNA occupancy (for non-ratcheted population only, step 8) and/or ArfB occupancy (step 9). The three resulting homogeneous ribosome particle populations were refined to high-resolution according to the gold-standard procedure and overall resolutions were determined using high-resolution noise substitution (Supplementary Fig. 2e).

For visualization and atomic model refinement all final maps were amplitude sharpened globally using PHENIX 1.16^55^. The two best-resolved cryo-EM maps—the P + 9 post-hydrolysis state at 2.6 Å and the P + 9 stalled complex at 3.1 Å—were resampled to a finer pixel size of 0.6525 Å for improved visualization and interpretation. We first created an atomic model for the highest-resolved 2.6 Å cryo-EM map. An initial model was built by rigid body fitting in ChimeraX 0.91^56^ based on the following structures: PDB 5AFI^57^ for the *E. coli* 70S ribosome, PDB 4RB7^58^ for tRNA^Phe^, PDB 5O2R^21^ for Api137 and PDB 4V95^15^ for ArfB. Parts of ArfB had to be re-built manually in WinCoot^59^ 0.8.9.2 to fit our high-resolution density due to register shifts with respect to the reported model from the 3.2 Å crystal structure of *E. coli* ArfB bound to the *Thermus thermophilus* ribosome (PDB 4V95); register shifts—by one amino acid—occurred in the N-terminal domain (Ile2 to His7, Thr33 to Ser46) and all residues of the C-terminal domain starting from Arg112. Metal coordination and secondary structure restraints were prepared using initial models with phenix.ready_set. Additional structural restraints were generated from hydrogen bond search in ChimeraX. Real space refinement was performed using phenix.real_space_refine with global minimization, simulated annealing, atomic displacement parameters and local grid search for 300 iterations limit and 5 macrocycles. Atomic model refinement of the remaining states (P + 0 post-hydrolysis state, P + 9 stalled complex and P + 9 hybrid state) was based on the model of the P + 9 post-hydrolysis state and performed in an analogous way. As an additional validation step we also refined the scrambled atomic models against one of the half maps and calculated FSCs of the resulting model against the second half map. To remove possible model bias, atomic models from the full-map refinement were scrambled by applying random shifts of 0.25 Å to all atomic positions beforehand. Modeling statistics are described in Table 1, FSC curves are depicted in Supplementary Fig. 2e.

### Reporting summary

Further information on research design is available in the Nature Research Reporting Summary linked to this article.

## Supplementary information

Supplementary Information

Peer Review

Reporting Summary

## Data Availability

Cryo-EM maps/associated coordinates of atomic models have been deposited in the Electron Microscopy Data Bank/Protein Data Bank with the following accession codes: PDB 6YSR and EMD-10905 (P + 9 stalled complex), PDB 6YSS and EMD-10906 (P + 9 post-hydrolysis), PDB 6YST and EMD-10907 (P + 9 tRNA hybrid state), PDB 6YSU and EMD-10908 (P + 0 post-hydrolysis). Cryo-EM micrographs and particle images have been deposited in the EMPIAR database (https://www.ebi.ac.uk/pdbe/emdb/empiar/) with accession code EMPIAR-10443. Source data are provided with this paper.

## Source data

Source Data
