# Supplementary material for: Mechanism of ribosome rescue by alternative ribosome-rescue factor B
Source: Nat Commun. 2020 Aug 14;11:4106. doi: 10.1038/s41467-020-17853-7 (PMC7427801; doi:10.1038/s41467-020-17853-7)
Supplement: Supplementary file 1 — Supplementary Information [file 41467_2020_17853_MOESM1_ESM.pdf]

Mechanism of Ribosome Rescue by Alternative Ribosome-Rescue Factor B - **Supplementary Information**

Kai-Hsin Chan[1], Valentyn Petrychenko[2], Claudia Mueller[3], Cristina Maracci[1], Wolf Holtkamp[1,4], Daniel N. Wilson[3], Niels Fischer[2*] and Marina V. Rodnina[1*]

[1] Department of Physical Biochemistry, Max Planck Institute for Biophysical Chemistry, Am Fassberg 11, 37077 Göttingen, Germany

[2] Department of Structural Dynamics, Max Planck Institute for Biophysical Chemistry, Am Fassberg 11, 37077 Göttingen, Germany

[3] Institute for Biochemistry and Molecular Biology, University of Hamburg, Martin-Luther-King-Platz 6, 20146 Hamburg, Germany

[4] Present address: Paul-Ehrlich-Institut, Paul-Ehrlich-Straße 51-59, 63225 Langen

* Correspondence to: niels.fischer@mpibpc.mpg.de (ORCID 0000-0002-4609-4052) and rodnina@mpibpc.mpg.de (ORCID 0000-0003-0105-3879)

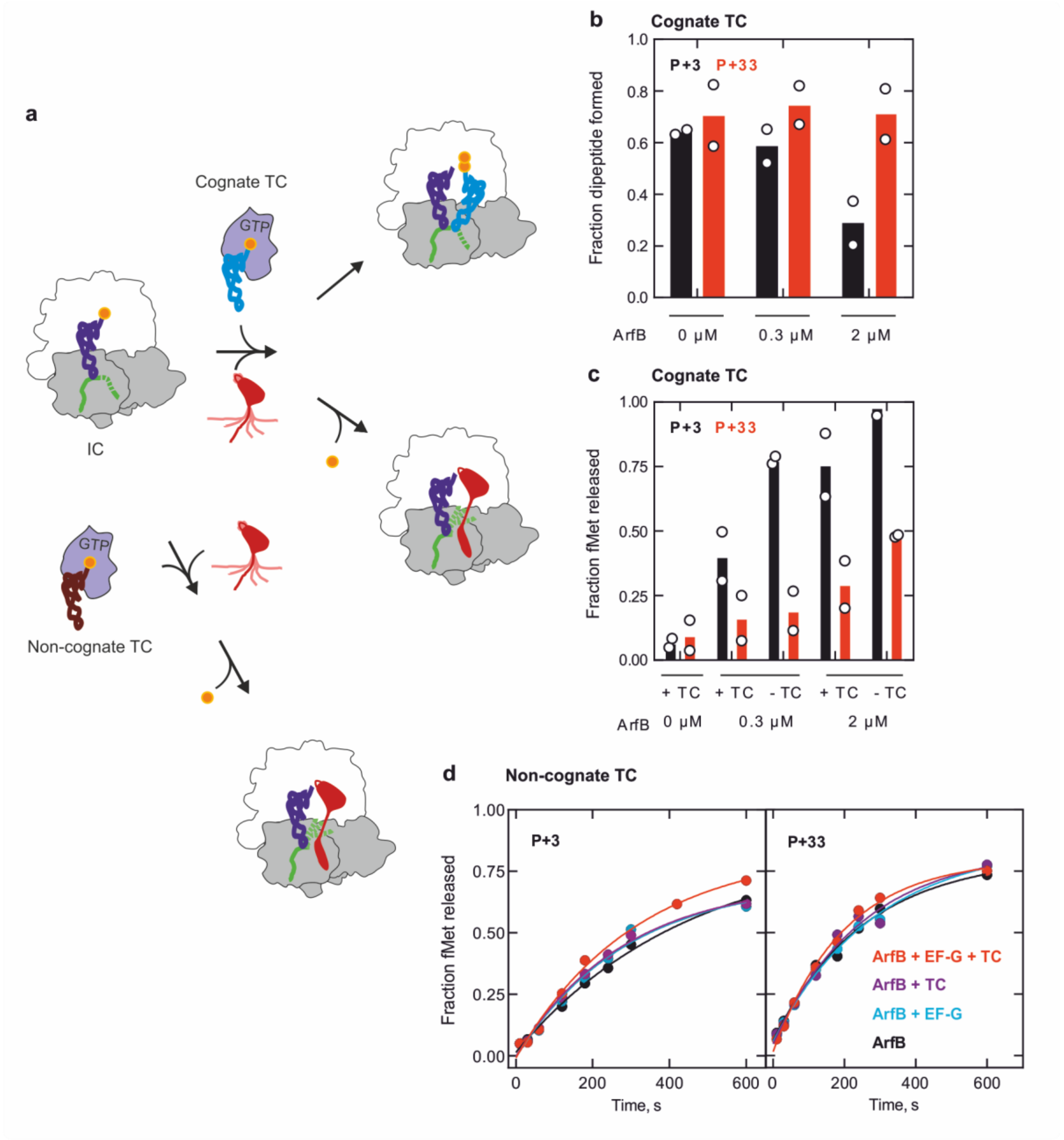

Supplementary Figure 1 | Cognate ternary complexes (TC), but not non-cognate TC, compete with ArfB for binding to the ribosome. a, Experimental assay to measure competition between ternary complexes and ArfB on ribosome complexes with P+3 or P+33 mRNAs and [^{3}H]fMet-tRNA in the P site (IC). TC activity is monitored by dipeptide formation (f[^{3}H]Met-[^{14}C]Phe), ArfB activity by f[^{3}H]Met release from f[^{3}H]Met-tRNA^{fMet}. **b, c,** Dipeptide formation (**b**) and ArfB-induced fMet release (**c**) on P+3 and P+33 initiation complex (IC, 0.5 µM) mixed with cognate EF-Tu–GTP–[^{14}C]Phe-tRNA^{Phe} (0.25 µM), and ArfB (0.3 or 2 µM). Data are presented as mean values from two biological replicates (white circles). **d**, ArfB-induced fMet-tRNA hydrolysis on P+3 and P+33 IC (0.5 µM) in the presence of ArfB (0.1 µM and 2 µM, respectively), non-cognate EF-Tu–GTP–Val-tRNA^{Val} (5 µM) and EF-G (2 µM).

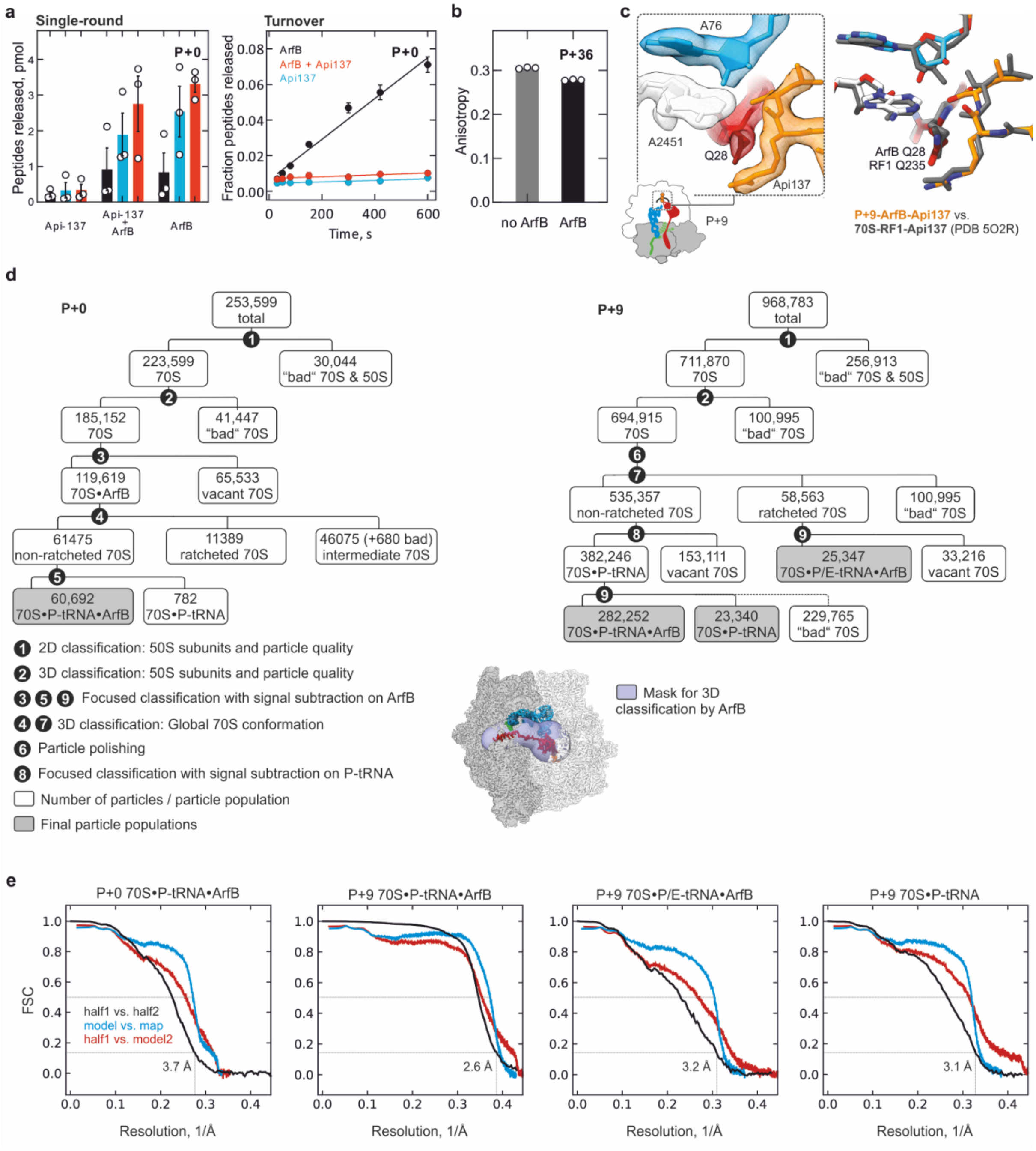

Supplementary Figure 2 | Cryo-EM analysis of Api137-stalled ribosome–ArfB complexes. a, Api137 traps ArfB on stalled ribosomes after one round of hydrolysis. Left: Single-round peptidyl-tRNA hydrolysis of P+0 complexes (0.1 µM) by ArfB (1 µM) in the presence or absence of Api137 (1 µM). Right: Multiple-turnover peptidyl-tRNA hydrolysis on P+0 complexes (0.2 µM) by ArfB (0.02 µM) in the presence or absence of Api137 (1 µM). Error bars represent the SEM of three biological replicates. **b,** mRNA remains intact after incubation with ArfB. Fluorescence anisotropy of fluorescein attached at the 3' end of ribosome-bound P+36 mRNA is shown before and after addition of ArfB (0.1 µM) to P+36 ribosome complex (0.01 µM). Error bars represent the SEM of three biological replicates (white circles). **c,** Api137 stalls ArfB and canonical release factors on the ribosome by a similar structural

mechanism. Left: Cryo-EM density of the PTC in the major P+9 post-hydrolysis state, rendered at 3.5σ. Right: Corresponding interaction network of Api37 and the ribosome with ArfB vs. the network reported for RF1[1]. **d,** Sorting of P+0 complexes (left) and P+9 complexes (right) and the mask used for 3D classification for ArfB occupancy (lower right, transparent blue). See Methods for further details. **e,** Fourier-shell-correlation (FSC) curves of P+0 complexes (left) and P+9 complexes.

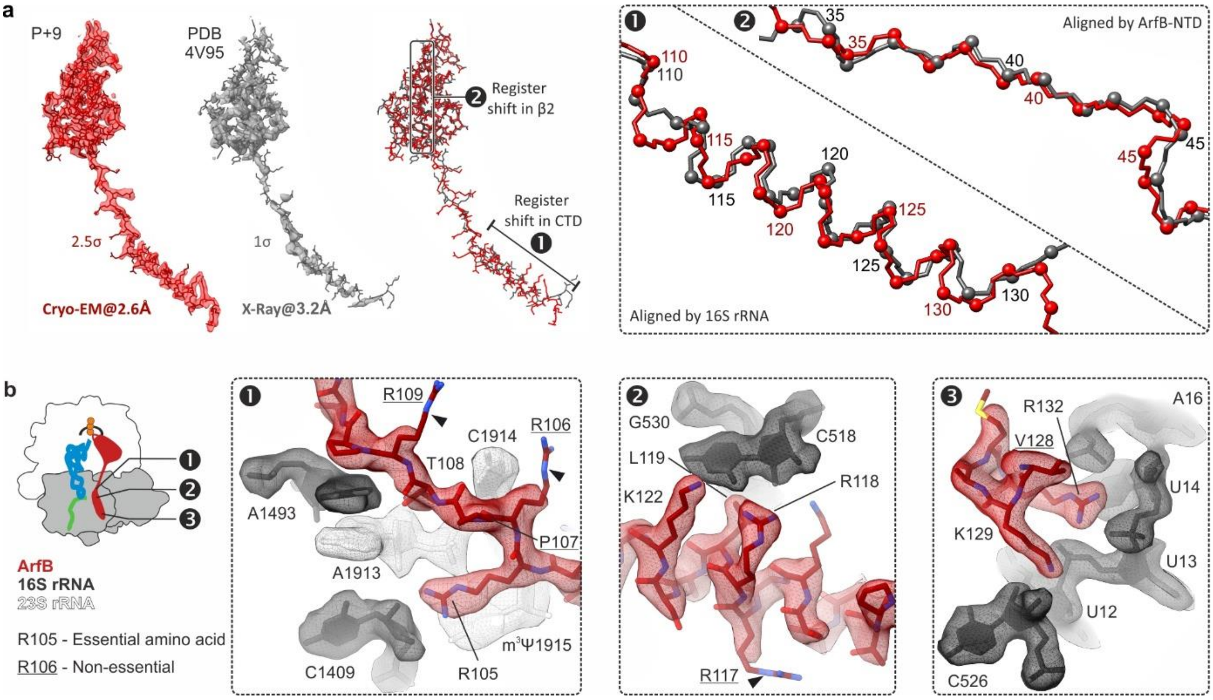

Supplementary Figure 3 | Quality of the ArfB models. **a,** Improved atomic model of the ribosome-ArfB complex from the present high-resolution 2.6 Å cryo-EM map. Left: Present cryo-EM density and model of ArfB in the P+9 post-hydrolysis complex, density rendered at 3.5σ. Middle: X-ray based density ($2mF_0$-DF_c) and corresponding model of ArfB in the 3.2 Å crystal structure of a post-hydrolysis complex[2], density rendered at 1σ. Right: Superposition of the cryo-EM and X-ray based models reveals register shift between the models. Box: Close-ups of register shifts between the models for backbone regions as indicated by labels 1, 2 in the superposition; Cα atoms are rendered as spheres with residue numbers. The register shift in the CTD starting at Arg112 affects all subsequent residues and their interactions with the ribosome. **b**, Cryo-EM densities for functionally important ArfB residues. Note the well-defined density for ArfB residues forming important interactions vs. the undefined densities (arrow-heads) for non-essential residues (underlined), which are not involved in tight interactions, in accordance with the functional data[3]. Panels 1, 2, 3 depict distinct regions in ArfB indicated in the schematic (left).

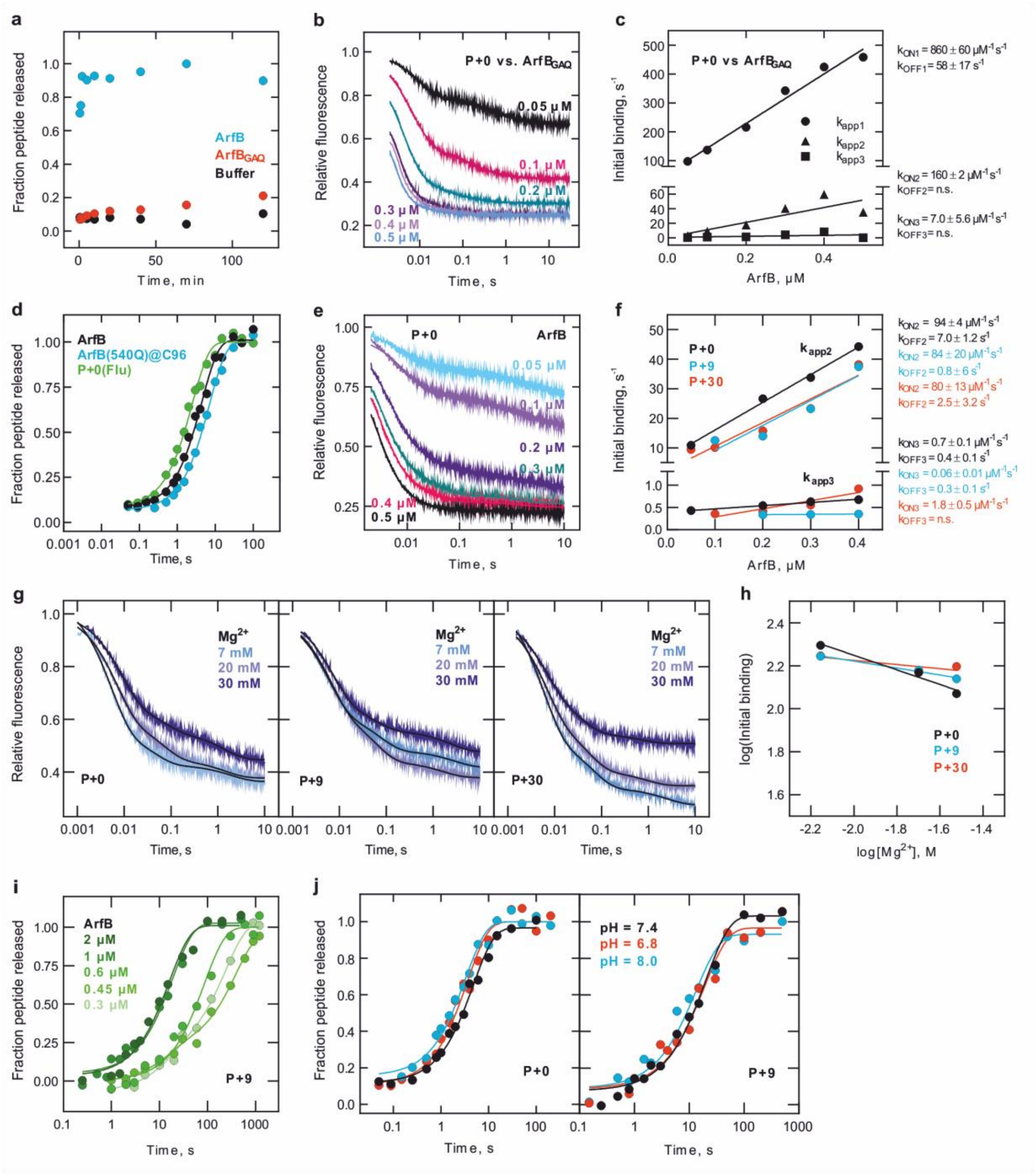

Supplementary Figure 4 | Detailed characterization of ArfB initial binding and engagement steps. a, $ArfB_{GAQ}$ is catalytically inactive at 20°C. ArfB, $ArfB_{GAQ}$ (1 µM) or buffer were mixed with P+0 complex (0.1 µM) and time courses of peptidyl-tRNA hydrolysis were measured. **b,** Time courses of initial binding are similar for $ArfB_{GAQ}$ and the catalytically active wild-type ArfB (compare to **e**). $ArfB_{GAQ}$(540Q) (0.05-0.5 µM) was rapidly mixed with P+0(Flu) complexes (0.015 µM). **c,** Linear concentration dependence of the apparent rate constants for the binding of $ArfB_{GAQ}$ to P+0. Data are represented as mean values of two independent experiments with six technical replicates each. **d,** Labeled components used in binding experiments have the same activity in the peptidyl-tRNA hydrolysis

reaction as unlabeled wild-type ArfB. Time courses of peptidyl-tRNA hydrolysis with the wt ArfB or ArfB(540Q) labeled at position 96 (1 µM) on P+0 (0.15 µM), and of wt ArfB on P+0(Flu) complexes (37°C). Solid lines represent single-exponential fits. **e,** Time courses of ArfB binding to P+0. P+0(Flu) (0.015 µM) was rapidly mixed with ArfB(540Q) (0.05-0.5 µM). Solid lines indicate exponential fits with three exponential terms. **f,** Linear concentration dependence of the apparent rate constants for the intermediate and slow phases of ArfB binding to P+0, P+9, and P+30 complexes. **g,** Rapid initial binding of ArfB to the ribosome at increasing Mg^{2+} concentrations. ArfB(540Q) (0.2 µM) was rapidly mixed with P+0(Flu), P+9(Flu), or P+30(Flu) (0.015 µM) at 7 mM, 20 mM, or 30 mM $MgCl_2$. **h,** Effect of Mg^{2+} on the apparent rate constant of the rapid association phase. Data represented as mean values of two independent experiments with six technical replicates each. **i,** Kinetics of peptidyl-tRNA hydrolysis on P+9 complexes. Time courses of hydrolysis with P+9 ribosome complex (0.15 µM) and ArfB (0.3-2 µM) (37°C). Data are represented as mean values of two independent experiments. **j,** ArfB-mediated peptidyl-tRNA hydrolysis is pH-independent. Time courses were measured at pH = 7.4, 6.8, or 8.0 with P+0 (left panel) and P+9 (right panel) complexes. ArfB (1 µM) was rapidly mixed with stalled ribosomes (0.15 µM) at 37°C.

Supplementary Table 1. Cryo-EM structure determination.

**Ribosomal complex**	P+0 70S•tRNA•ArfB•Api137	P+9 70S•tRNA•ArfB•Api137	P+9 70S•Phe-fMet-tRNA	P+9 70S•tRNA•ArfB•Api137
Ribosomal state	P+0 post-hydrolysis	P+9 post-hydrolysis	P+9 stalled complex	P+9 tRNA hybrid state
**Database entries**				
EMDB ID	10908	10906	10905	10907
PDB ID	6YSU	6YSS	6YSR	6YST
**Data collection**				
Microscope	Titan Krios	Titan Krios	Titan Krios	Titan Krios
Camera	Falcon II	Falcon III	Falcon III	Falcon III
Magnification	59.000	59.000	59.000	59.000
Voltage (kV)	300	300	300	300
Electron dose ($e^-/Å^2$)	25	50	50	50
Defocus range (μm)	1.2-2.3	0.2-2.5	0.2-2.5	0.2-2.5
Pixel size (Å)	1.072	1.16	1.16	1.16
**Cryo-EM reconstruction**				
Final resolution	3.7	2.6	3.1	3.2
Final particles (no.)	60.692	282.252	25.347	23.340
Point group symmetry	*C1*	*C1*	*C1*	*C1*
FSC-threshold	0.143	0.143	0.143	0.143
Resolution (Å)	3.7	2.6	3.1	3.2
Resolution metric	gold standard FSC	gold standard FSC	gold standard FSC	gold standard FSC
**Atomic model refinement**				
Final resolution (Å)	3.7	2.6	3.1	3.2
Cumulative RSCC (%) >0.8/>0.6/>0.4	84.80%/96.49%/98.79%	87.47%/95.28%/97.23%	80.50%/94.66%/97.31%	67.52%/93.74%/97.93%
Initial models used	5afi (70S)	5afi (70S)	5afi (70S)	5afi (70S)
	4v95 (ArfB)	4v95 (ArfB)	4RB7 (P-tRNA)	4v95 (ArfB)
	5O2R (Api137)	5O2R (Api137)		5O2R (Api137)
	4RB7 (P-tRNA)	4RB7 (P-tRNA)		4RB7 (P-tRNA)
Molprobity score	2.41	2.05	2.15	2.45
Clashscore	20.23	10.58	12.11	20.46
**No. Atoms/No. Residues/RSCC**				
Total	146404/10675/0.85	147051/10751/0.86	145963/10607/0.83	146850/10748/0.81
Protein	46328/6030/0.84	46921/6105/0.86	45722/5954/0.82	46921/6105/0.78
Nucleic	99717/4645/0.87	99739/4646/0.87	99903/4653/0.85	99672/4643/0.83
ArfB	1078/140/0.75	1078/140/0.88	-	1078/140/0.84
**B-factors**				
Protein	77.38	36.20	63.65	96.08
Nucleotide	81.47	36.82	64.24	93.91
Ligands, Ions	54.69	23.40	43.02	60.77
**R.m.s. deviations**				
Bond lengths (Å)	0.007	0.007	0.006	0.007
Bond angles (°)	0.758	0.685	0.737	0.847
**Ramachandran plot**				
Favored (%)	87.72	91.50	89.74	86.10
Allowed (%)	12.10	8.08	9.88	13.49
Disallowed (%)	0.19	0.42	0.38	0.42

*For model refinement, maps at ≤3.1Å resolution were resampled to 512×512×512 pixels, corresponding to a pixel size of 0.6525Å

Supplementary Table 2. Summary of rate constants.

	P+0	P+9	P+30
Hydrolysis rate, s^{-1}	0.15 ± 0.01	0.06 ± 0.01	0.004 ± 0.001
Fast k_{ON}, $\mu M^{-1}s^{-1}$	470 ± 70	280 ± 30	320 ± 40
Fast k_{OFF}, s^{-1}	110 ± 20	140 ± 10	120 ± 10
Medium k_{ON}, $\mu M^{-1}s^{-1}$	94 ± 4	84 ± 20	80 ± 13
Medium k_{OFF}, s^{-1}	7.0 ± 1.2	0.8 ± 6	2.5 ± 3.2
Slow k_{ON}, $\mu M^{-1}s^{-1}$	0.7 ± 0.1	0.06 ± 0.01	1.8 ± 0.5
Slow k_{OFF}, s^{-1}	0.4 ± 0.1	0.3 ± 0.1	n.s.
$k_{diss\ avg}$, s^{-1}	0.06 ± 0.01	0.23 ± 0.01	0.33 ± 0.01
K_M, µM	0.25 ± 0.09	n.s.	
k_{cat}, s^{-1}	0.010 ± 0.001	n.s.	

The hydrolysis rate is obtained by exponential fitting of single-round hydrolysis time courses (Fig. 4d,e). The k_{ON} and k_{OFF} values were calculated from the slope and Y-axis intercept, respectively, of the linear concentration dependence of the apparent rate constant of the rapid initial binding phase (Fig. 4c). k_{diss_avg} is a weighted average dissociation rate constant of the ArfB-ribosome complex estimated from the time courses of Fig. 4f. K_M is the substrate concentration at which the reaction velocity reaches half-maximum and is calculated by hyperbolic fitting of the Michaelis-Menten titration (Fig. 5A). k_{cat} is calculated by dividing the maximum reaction velocity with the ArfB concentration in the reaction.

Supplementary Table 3. Apparent rate constants of ArfB dissociation.

	k_{app}^1, s^{-1}	A_1	k_{app}^2, s^{-1}	A_2
**P+0**	0.41 ± 0.01	0.21 ± 0.02	0.04 ± 0.01	0.79 ± 0.02
**P+9**	0.41 ± 0.01	0.47 ± 0.01	0.07 ± 0.01	0.53 ± 0.01
**P+30**	0.47 ± 0.01	0.61 ± 0.01	0.10 ± 0.01	0.39 ± 0.01

Apparent rate constants were obtained from the exponential fit of representative dissociation experiments with at least 6 technical replicates averaged. Values are averages with SEM of the fit.

Supplementary References

1. Florin, T. et al. An antimicrobial peptide that inhibits translation by trapping release factors on the ribosome. *Nat Struct Mol Biol* **24**, 752-757 (2017).
2. Gagnon, M.G., Seetharaman, S.V., Bulkley, D. & Steitz, T.A. Structural basis for the rescue of stalled ribosomes: structure of YaeJ bound to the ribosome. *Science* **335**, 1370-2 (2012).
3. Kogure, H. et al. Identification of residues required for stalled-ribosome rescue in the codon-independent release factor YaeJ. *Nucleic Acids Res* **42**, 3152-63 (2014).
