# Supplementary material for: Mechanism of ribosome rescue by alternative ribosome-rescue factor B
Source: Nat Commun. 2020 Aug 14;11:4106. doi: 10.1038/s41467-020-17853-7 (PMC7427801; doi:10.1038/s41467-020-17853-7)
Supplement: Supplementary file 2 — Peer Review [file 41467_2020_17853_MOESM2_ESM.pdf]

Reviewer #1 (Remarks to the Author):

Chen et al. describes several biochemical and kinetic studies to decipher the mode of action of the alternative ribosome-rescue factor ArfB along with presenting several high resolution cryo-EM structures of ArfB bound to a model non-stop mRNA ribosome complex. The presented body of work is quite comprehensive and provides important data helping to rule out other proposed functional roles of ArfB in the cell, i.e. rescuing ribosomes stalled on rare codon clusters in an mRNA. Furthermore, the authors can put forward a mechanism for ArfB-mediated ribosome rescue considering several previously unknown kinetic steps found in their thorough kinetic analysis to provide the most comprehensive understanding of ArfB function to date. The cryo-EM models presented in this manuscript showcases the full ArfB protein interacting with the ribosome including improved resolution for the flexible linker between the N- and C-terminal domains that improve the biochemical interpretation based on the earlier crystal structure of this complex (DOI: DOI: 10.1126/science.1217443). The resolution of the linker domain provided insight into how the dynamic nature of ArfB contributes to its functional mechanism in the absence of other termination factors like RF2 and that is required for the function of alternative ribosome rescue factor ArfA. Additionally, the resolution of the linker domain revealed why the mutation of residues in the linker resulted in abolished ArfB activity as described by Kogure et al. (DOI: 10.1093/nar/gkt1280). Overall, the cryo-EM models and the impressive kinetic analysis of ArfB-mediated ribosome rescue presented here further our understanding of ArfB not only in bacteria, but as to how the eukaryotic mitochondrial targeted ortholog, ICT1, may function to rescue ribosomes stuck on a truncated, non-stop mRNA. As such this manuscript is of general interest, providing important structural and mechanic understanding beyond the bacterial ribosome and is well suited be published in nature communication.

Major Issues
No major issues.

Minor Issues
I found the paragraph at the end of the results (line 232 – 252) slightly confusing containing some hard to follow leaps in logic. The dissociation rate constant for the experiment described in this paragraph derives from the data (Fig 4f) fit with an exponential curve, or by some other means? (Line 237 – in the range of <1 s-1 at 20°C). In the subsequent sentence, lines 239-240 specifically, it is stated that the data in Fig. 4b is fit with a two-exponential equation, and that two rate constants where derived for each complex. It is then concluded that the real difference between the complexes is in the "fraction of the slow vs. fast steps," (Line 241-243), which I assume is the proportion of the amplitude change attributed to each rate constant from the fit of the curve? Is the overall conclusion from the fast vs slow steps that there are two steps in the dissociation of ArfB from the ribosome, or that there are two populations of ArfB-ribosome complexes with one rate of dissociation each? It might benefit the reader to see which step in the proposed mechanism is being referred to by the "preceding step" (line 245).
Furthermore, given that the dynamic properties if the linker and the C-terminal domain are critical for the specificity and selection described in the model it would be important to discuss the thermodynamic details / implications that such a model would have (e.g. with respect to enthalpic vs entropic contributions). (maybe around line 310 or/and 351)
The discussion might also provide some more detail about the mechanism of ArfB dissociation based on the kinetic data reported by the authors (maybe around line 340)
Line 179 – the word binding is in a different font, could just be the PDF proof.
Line 198 – "Supplementary fig. e,f)." – no figure number not listed, just panel e,f. I assume sup. Fig. 4
Line 210 – why using different precision indicators for koff e.g. ~110s-1 vs. 110-140s-1.
Line 345 – How do the authors envision mechanistically such a "guiding" process?
Line 393 – Missing a period at the end of the sentence "…at 37°C."
Line 458 – Should ArfB[flu]-96C be ArfB[Flu]-96C?
Line 754 – Fig 3e some means of precision / significance indication (e.g. error bars) should be

provided to allow assessment of the differences between the values?
Line 770 – There is a period after the | divide between "Supplementary Fig. 3" and "Quality of the ArfB models."
Line 776 – Are the authors wanting to have alpha spelled out or with the Greek letter?

Reviewer #2 (Remarks to the Author):

Review -"Mechanism of Ribosome Rescue by Alternative Release Factor B"

Kai-Hsin Chan and coauthors have studied by biochemical and structural methods the multistep mechanism of ArfB action of ribosome rescue. Authors significantly expand the X-ray studies of ArfB bound to ribosome (T. Steiz lab). Presented data shows a kinetic profile of ArfB action with structural snapshots of several steps.
The paper is a good example of an integrative approach of biochemical and structural to study of the problem.
The paper is well written and I would like to recommend for publication in Nature Communication.

Some comments:
Autors suggested: "Mechanism of ribosome rescue by ArfB based on rapid kinetics and cryo-EM data". One of the most important model in the mechanism is intermediate state - initial binding of ArfB to the ribosome which is based on the NMR ensemble of free ArfB and cryo-EM structures of the P+9 ribosome-ArfB complex. This is not an experimental intermediate state complex. I would like to ask authors to discuss about possibility to form complex containing simultaneously mRNA P+99 and ArfB or mRNA P+99 and ArfB mutant without CTD domain.

Reviewer #3 (Remarks to the Author):

The manuscript by Chan et al. "Mechanism of ribosome rescue by alternative release factor B" provides kinetics and structural insights into the mechanism of ribosome rescue by ArfB. The authors use rapid kinetics to clarify previous findings that suggested that ArfB can act on ribosome stalled on long mRNAs, including those containing rare codons. The authors show that ArfB-mediated ribosome rescue more likely occurs on mRNAs with no or short mRNA overhangs extending downstream of the P site. The efficiency of ribosome rescue by ArfB decreases rapidly on longer mRNAs. The data suggest that ArfB first binds to the stalled ribosome independently of the length of the mRNA, and that the following accommodation step is then dependent on the presence of mRNA in the mRNA entry channel. Using cryo-EM, the authors visualize ArfB bound to stalled ribosome complexes in two major states: one with no A site overhang, and one with a 9-nucleotide overhang. The 9-nucleotide overhang structure shows that ArfB displaces the 3' end of the mRNA from the mRNA entry channel, which agrees with the slower accommodation step on ribosome complexes with longer mRNAs. In both structures, the C-terminal tail of ArfB is bound in the mRNA entry channel, making it incompatible with longer mRNAs. This observation is in agreement with a previous crystal structure of YaeJ (ArfB) bound to the Thermus thermophilus 70S ribosome. Interestingly, the high-resolution cryo-EM reported here (2.6 Å) allows the authors to build a more accurate model for ArfB which appears to better explain the available mutational data. The work is well executed and the manuscript is clearly written. This reviewer has only minor comments and suggestions to improve the manuscript:

Lines 86-87: Change the order of the sentence to "When more than 9 nts of the mRNA extended past the P-site codon, the rate of hydrolysis reaction decreases sharply, by about…"

Lines 152-155: Could be clearer to change to “In agreement with mutational data, the cryo-EM-based model shows that the essential residues Arg105, Arg118, Leu119, Lys122, Lys129, and Arg132 form intricate side chain contacts with the ribosomal RNA (rRNA), whereas most non-essential side chains that are not involved in any interaction are less well-resolved.”

Line 252: “…(Fig. 4g).” – There is no panel g in figure 4.

Line 325: Could be clearer to write “…when the mRNA exceeds the P-site codon by more than 6 nts.”

Line 346: “…to adopt to different…” Remove extra word, or different meaning?

Line 773: “…3.1 Å crystal structure…” This is a 3.2 Å structure. Same comment in panel a, label underneath the gray structure.

Reviewer #1 - Remarks to the Authors

Chen et al. describes several biochemical and kinetic studies to decipher the mode of action of the alternative ribosome-rescue factor ArfB along with presenting several high resolution cryo-EM structures of ArfB bound to a model non-stop mRNA ribosome complex. The presented body of work is quite comprehensive and provides important data helping to rule out other proposed functional roles of ArfB in the cell, i.e. rescuing ribosomes stalled on rare codon clusters in an mRNA. Furthermore, the authors can put forward a mechanism for ArfB-mediated ribosome rescue considering several previously unknown kinetic steps found in their thorough kinetic analysis to provide the most comprehensive understanding of ArfB function to date. The cryo-EM models presented in this manuscript showcases the full ArfB protein interacting with the ribosome including improved resolution for the flexible linker between the N- and C-terminal domains that improve the biochemical interpretation based on the earlier crystal structure of this complex (DOI: DOI: 10.1126/science.1217443). The resolution of the linker domain provided insight into how the dynamic nature of ArfB contributes to its functional mechanism in the absence of other termination factors like RF2 and that is required for the function of alternative ribosome rescue factor ArfA. Additionally, the resolution of the linker domain revealed why the mutation of residues in the linker resulted in abolished ArfB activity as described by Kogure et al. (DOI: 10.1093/nar/gkt1280). Overall, the cryo-EM models and the impressive kinetic analysis of ArfB-mediated ribosome rescue presented here further our understanding of ArfB not only in bacteria, but as to how the eukaryotic mitochondrial targeted ortholog, ICT1, may function to rescue ribosomes stuck on a truncated, non-stop mRNA. As such this manuscript is of general interest, providing important structural and mechanic understanding beyond the bacterial ribosome and is well suited be published in nature communication.

Reply: We thank the reviewer for the very positive evaluation of our work and for the helpful suggestions.

Reviewer #1 - Major Issues

No major issues.

Reviewer #1 - Minor Issues

1. *I found the paragraph at the end of the results (line 232 – 252) slightly confusing containing some hard to follow leaps in logic. The dissociation rate constant for the experiment described in this paragraph derives from the data (Fig 4f) fit with an exponential curve, or by some other means? (Line 237 – in the range of <1 s-1 at 20°C). In the subsequent sentence, lines 239-240 specifically, it is stated that the data in Fig. 4b is fit with a two-exponential equation, and that two rate constants where derived for each complex. It is then concluded that the real difference between the complexes is in the "fraction of the slow vs. fast steps," (Line 241-243), which I assume is the proportion of the amplitude change attributed to each rate constant from the fit of the curve? Is the overall conclusion from the fast vs slow steps that there are two steps in the dissociation of ArfB from the ribosome, or that there are two populations of ArfB-ribosome complexes with one rate of dissociation each? It might benefit the reader to see which step in the proposed mechanism is being referred to by the "preceding step" (line 245).*

Reply: The dissociation rate constants mentioned in former lines 233-235 are derived from exponential fitting described in the following sentence. To make it clearer, we have moved this sentence to below (lines 251-253 in the revised version) where it provides the comparison between the rates of dissociation of the engaged complex with the initial binding complex. The fraction of

slow dissociating and fast dissociating ArfB is indeed inferred from the amplitude of fluorescence change for each step, and a sentence has been added to line 243-244 to explain this. We also clarified in the text what we meant as a "preceding step". As there are two alternatives to explain the two exponential steps, as we discuss in lines 245-251, we would prefer not to indicate this in the scheme, as it would reduce the visual clarity.

2. *Furthermore, given that the dynamic properties if the linker and the C-terminal domain are critical for the specificity and selection described in the model it would be important to discuss the thermodynamic details / implications that such a model would have (e.g. with respect to enthalpic vs entropic contributions). (maybe around line 310 or/and 351).*

Reply: A sentence speculating on the possible enthalpy-entropy compensation of the binding and folding of the C-terminal tail has been added (lines 313-314 in the revised version): "The favorable enthalpic contribution of forming specific interactions with the ribosome may be offset by the entropic cost of folding of the C-terminal domain."

3. *The discussion might also provide some more detail about the mechanism of ArfB dissociation based on the kinetic data reported by the authors (maybe around line 340).*

Reply: We thank the reviewer for this idea and added a sentence (lines 340-342) discussing the possibility that the dissociation rate includes both unbinding and unfolding of the C-terminal tail: "The tight interactions that persist after peptide hydrolysis likely contribute to the slow dissociation rate, which may encompass the rates for unbinding as well as unfolding of the ArfB C-terminal domain.."

Line 179 – *the word binding is in a different font, could just be the PDF proof.*

Reply: We changed font from Times New Roman to Calibri.

Line 198 – *"Supplementary fig. e,f)." – no figure number not listed, just panel e,f. I assume sup. Fig. 4*

Reply: Changed to Supplementary Fig. 4 e,f (now in line 197).

Line 210 – *why using different precision indicators for koff e.g. ~110s-1 vs. 110-140s-1.*

Reply: For clarity, we have edited the sentence to read "The binding is very rapid with k_{ON} of ~500 $\mu M^{-1}s^{-1}$ for P+0 and ~300 $\mu M^{-1}s^{-1}$ for P+9 and P+30. The initial binding complex is also labile, with k_{OFF} of ~110 s^{-1}, ~140 s^{-1}, and ~120 s^{-1} for P+0, P+9, and P+30, respectively, which shows that the initial binding is independent of the mRNA length."

Line 345 – *How do the authors envision mechanistically such a "guiding" process?*

Reply: This is now better explained (in lines 316-318): "possibly guided by electrostatic steering by the acidic phosphate backbone of the ribosome".

Line 393 – *Missing a period at the end of the sentence "…at 37°C."*

Reply: Period now inserted.

Line 458 – *Should ArfB[flu]-96C be ArfB[Flu]-96C?*

Reply: Yes, changed accordingly.

Line 754 – *Fig 3e some means of precision / significance indication (e.g. error bars) should be provided to allow assessment of the differences between the values?*

Reply: Error bars from replicates have been added to the figure and the legend changed accordingly (line 774).

Line 770 – *There is a period after the | divide between "Supplementary Fig. 3" and "Quality of the ArfB models."*

Reply: Removed.

Line 776 – *Are the authors wanting to have alpha spelled out or with the Greek letter?*

Reply: Changed to greek letter.

Reviewer #2 - Remarks to the Authors

Kai-Hsin Chan and coauthors have studied by biochemical and structural methods the multistep mechanism of ArfB action of ribosome rescue. Authors significantly expand the X-ray studies of ArfB bound to ribosome (T. Steiz lab). Presented data shows a kinetic profile of ArfB action with structural snapshots of several steps. The paper is a good example of an integrative approach of biochemical and structural to study of the problem. The paper is well written and I would like to recommend for publication in Nature Communication.

Reply: We thank the reviewer for the positive evaluation of our work.

Reviewer #2 – Comments

Autors suggested: "Mechanism of ribosome rescue by ArfB based on rapid kinetics and cryo-EM data". One of the most important model in the mechanism is intermediate state - initial binding of ArfB to the ribosome which is based on the NMR ensemble of free ArfB and cryo-EM structures of the P+9 ribosome-ArfB complex. This is not an experimental intermediate state complex. I would like to ask authors to discuss about possibility to form complex containing simultaneously mRNA P+99 and ArfB or mRNA P+99 and ArfB mutant without CTD domain.

Reply: The structural model for the initial binding of ArfB based on the NMR structures is part of the discussion and is clearly a hypothesis, albeit consistent with all our kinetic data and previous mutational analysis. To make this clearer, we changed the word "infer" to "propose" in the sentence that introduces the model (line 284 in revised version). The activity of ArfB on mRNA +30 is almost as low as on +99, which is why we used mRNA+30 as a model for a long mRNA. We show that ArfB forms the initial binding and the engaged complex with P+30 ribosomes (Fig. 4c,f and Supplementary Fig. 4c,f). These findings indicate that the complex – if used in cryo-EM analysis – would not represent the dynamics of the initial binding complex alone, and thus is unlikely to provide more structural information than we show for P+9. Alternatively, if ArfB binding to the ribosome is not stabilized by the engagement, the complexes are likely just too unstable (k_{OFF} ~100 s^{-1}) to be analyzed by cryo-EM. Same is true for the complexes with isolated ArfB domains: if the complex is not stabilized by the interactions with the CTD, it is most likely too unstable to be captured by cryo-EM. Thus, at the moment structural studies on the initial binding complex are not feasible.

Reviewer #3 - Remarks to the Authors

The manuscript by Chan et al. "Mechanism of ribosome rescue by alternative release factor B" provides kinetics and structural insights into the mechanism of ribosome rescue by ArfB. The authors

use rapid kinetics to clarify previous findings that suggested that ArfB can act on ribosome stalled on long mRNAs, including those containing rare codons. The authors show that ArfB-mediated ribosome rescue more likely occurs on mRNAs with no or short mRNA overhangs extending downstream of the P site. The efficiency of ribosome rescue by ArfB decreases rapidly on longer mRNAs. The data suggest that ArfB first binds to the stalled ribosome independently of the length of the mRNA, and that the following accommodation step is then dependent on the presence of mRNA in the mRNA entry channel. Using cryo-EM, the authors visualize ArfB bound to stalled ribosome complexes in two major states: one with no A site overhang, and one with a 9-nucleotide overhang. The 9-nucleotide overhang structure shows that ArfB displaces the 3' end of the mRNA from the mRNA entry channel, which agrees with the slower accommodation step on ribosome complexes with longer mRNAs. In both structures, the C-terminal tail of ArfB is bound in the mRNA entry channel, making it incompatible with longer mRNAs. This observation is in agreement with a previous crystal structure of YaeJ (ArfB) bound to the Thermus thermophilus 70S ribosome. Interestingly, the high-resolution cryo-EM reported here (2.6 Å) allows the authors to build a more accurate model for ArfB which appears to better explain the available mutational data. The work is well executed and the manuscript is clearly written. This reviewer has only minor comments and suggestions to improve the manuscript:

Reply: We thank the reviewer for the positive assessment of our work.

Reviewer #3 – Comments

Lines 86-87: *Change the order of the sentence to "When more than 9 nts of the mRNA extended past the P-site codon, the rate of hydrolysis reaction decreases sharply, by about…"*

Reply: Done, thank you for a good suggestion.

Lines 152-155: *Could be clearer to change to "In agreement with mutational data, the cryo-EM-based model shows that the essential residues Arg105, Arg118, Leu119, Lys122, Lys129, and Arg132 form intricate side chain contacts with the ribosomal RNA (rRNA), whereas most non-essential side chains that are not involved in any interaction are less well-resolved."*

Reply: Thank you, this was implemented too.

Line 252: *"…(Fig. 4g)." – There is no panel g in figure 4.*

Reply: The reviewer is right, the words "Fig. 4g" were deleted (now line 255 in the revised version).

Line 325: *Could be clearer to write "…when the mRNA exceeds the P-site codon by more than 6 nts."*

Reply: This is a very good suggestion, which we implemented in line 328 (former line 325).

Line 346: *"…to adopt to different…" Remove extra word, or different meaning?*

Reply: It was a misprint which is now corrected (line 351 now).

Line 773: *"…3.1 Å crystal structure…" This is a 3.2 Å structure. Same comment in panel a, label underneath the gray structure.*

Reply: Thanks, we changed the resolution to the correct value of 3.2 Å for both instances (line 730 now).
